# A Mitochondria‐Specific Nanomedicine for Synergistic Chemo‐Photothermal Therapy and Immunogenic Activation Against Breast Cancer

**DOI:** 10.1002/advs.202522226

**Published:** 2026-02-25

**Authors:** Min Li, Jiangqi Feng, Junyang Zhuang, Qingguo Zhong, Yanda Li, Yanzhuo Lv, Shaoteng Huang, Xiangyu Huang, Mingbo Zhang, Xiaofeng Cai, Yuxin Wang, Wenping Chen, Zhenyu Duan, Zhou Chen, Kui Luo, Ning Li

**Affiliations:** ^1^ Fujian Key Laboratory of Drug Target Discovery and Structural and Functional Research School of Pharmacy Fujian Medical University Fuzhou China; ^2^ Department of Chemistry University College London London UK; ^3^ School of Medicine Fuzhou University Fuzhou China; ^4^ Department of Radiology Huaxi MR Research Center (HMRRC) Institution of Radiology and Medical Imaging Frontiers Science Center for Disease‐Related Molecular Network State Key Laboratory of Biotherapy West China Hospital Sichuan University Chengdu China

**Keywords:** amphiphilic peptide dendrimer, immunogenic cell death, mitochondria‐specific, synergic therapies, transcytosis

## Abstract

The presence of “cold” tumors in the tumor site is characterized with poor immune cell infiltration and resistance to conventional cancer therapies. To address these challenges, we developed an amphiphilic peptide dendrimer‐based multifunctional drug delivery system for integrating photothermal therapy (PTT), chemotherapy, and immunogenic cell death (ICD) to overcome the limitations of single‐modality treatments. Efficient drug delivery via the nanoplatform is realized through improving circulation stability, facilitating accumulation and penetration in tumor tissues, promoting cellular uptake, and accelerating tumor microenvironment‐responsive drug release. The mitochondria‐targeting property is derived from a mitochondria‐specific photothermal agent and a lipophilic cationic structure in the amphiphilic dendrimer, and it allows selective mitochondrial accumulation and disruption of the mitochondrial function. In vivo and in vitro studies confirm that PTT synergizes with chemotherapy to significantly enhance therapeutic efficacy, effectively induce ICD to modulate the tumor microenvironment, and strengthen antitumor responses. This nanoplatform enhances drug delivery, induces mitochondrial dysfunction, and achieves potent therapeutic effects, therefore, it could be fine‐tuned to become versatile for combination cancer therapies.

## Graphical abstract

Peptide dendrimer–based nanoplatform with mitochondria‐targeting capability was developed for synergistic chemo‐photothermal therapy. The system promotes efficient transcytosis, induces mitochondrial dysfunction, triggers immunogenic cell death (ICD), and enhance antitumor immunity by promoting dendritic cell maturation and T‐cell activation, resulting in improved tumor ablation and metastasis inhibition. This approach offers a platform integrating subcellular targeting with combination therapy and ICD‐mediated immunotherapy.

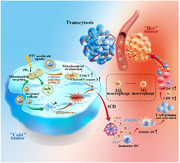

## 1 Introduction

The complexity and heterogeneity of the tumor microenvironment are the root causes for poor and inconsistent therapeutic responses during treating cancer via a single therapeutic modality, including chemotherapy and immunotherapy [1, 2, 3]. This complexity is compounded by the presence of “cold” tumors, which display limited immune cell infiltration and respond poorly to immune checkpoint therapies [3, 4]. The concept of immunogenic cell death (ICD) is emerging as a promising direction to overcome these challenges [5, 6]. ICD can transform a typically unresponsive “cold” tumor microenvironment into a “hot” microenvironment, allowing for the recruitment of immune cells into the tumor site. The tumor becomes susceptible to immunotherapies, leading to suppression of tumor growth, which may revolutionize cancer treatment [7, 8]. ICD is distinguished by the release of damage‐associated molecular patterns, represented by ATP, high mobility group box 1 (HMGB‐1), and calreticulin (CRT) [9]. They serve as immunostimulatory signals that facilitate dendritic cell–mediated antigen handling. Through this process, tumor‐associated antigens (TAAs) are efficiently processed and presented, leading to the priming of cytotoxic T lymphocytes (CTLs). Together, these events contribute to activate tumor‐specific anti‐tumor immune response [10, 11]. The induction of ICD can be facilitated through various therapeutic strategies, including chemotherapy [12], radiotherapy [13], and novel modalities including photothermal therapy [14, 15] and photodynamic therapy [16, 17, 18].

Among these modalities, chemotherapy remains a mainstay in cancer treatment, but there are major concerns over chemotherapy, including severe side effects, low water solubility, and poor tumor targeting of chemotherapeutic agents [19, 20]. Photothermal therapy (PTT), an emerging non‐invasive treatment modality for cancer, offers unique advantages such as high controllability, great precision, high efficacy, and low side effects to normal tissues [21, 22]. PTT has been shown to be effective in eliminating tumors through hyperthermia [23, 24]. However, single‐modality therapies are often ineffective in treating tumors with a diverse and complex nature because of their inherent limitations. For example, PTT‐alone treatment can cause incomplete tumor ablation due to inhomogeneous heat distribution within the tumor [25, 26, 27]. Synergistic combined therapy integrates multiple treatment modalities into a unified treatment strategy. By leveraging complementary mechanisms of action, this approach enhances overall therapeutic efficacy while addressing the shortcomings associated with monotherapy [28, 29]. Chemotherapy acts on a different mechanism from PTT. Systemic drug administration of chemotherapeutic agents can complement the localized effects induced by PTT through laser irradiation [30, 31]. The combination of PTT and chemotherapy has been commonly applied in clinical cancer treatment to eradicate cancer cells via a synergistic effect [32]. More importantly, both therapies can collaboratively induce ICD and amplify the therapeutic outcome by invoking immune‐mediated responses.

Cellular organelles, including mitochondria, and endoplasmic reticulum (ER), are essential for supporting cellular metabolic functions and maintaining homeostasis balance between cell proliferation and apoptosis [33, 34]. These organelles, with their heightened sensitivity, have become a focal point for targeted therapeutic strategies, which are garnering increasing research attention due to their potential for precision medicine [35, 36]. For instance, by harnessing the mitochondrial sensitivity to heat and reactive oxygen species, Jiang et al. [37] designed mitochondria‐targeting peptide‐based dendrimers and encapsulated them in liposomes to enhance mitochondrial targeting and enrichment efficiency of the delivery platform, resulting in a significant improvement in antitumor effects. The Tang's group [38] developed a strategy to combine aggregation‐induced emission (AIE) materials with ER‐targeting moieties. This approach stimulates an efflux of calcium ions from the ER, leading to depolarization of the ER membrane potential, which in turn induced ER stress along with crosstalk of the ER with mitochondria. This results in increased levels of pro‐apoptotic proteins such as Bax and CHOP, effectively triggering apoptosis in cancer cells. These organelle‐targeting strategies not only precisely modulate the drug transfer process from the plasma membrane to the target site to enhance the drug delivery efficiency, but also induce other effects such as apoptosis [39]. Therefore, organelle‐targeting therapeutic strategies hold significant clinical potential in reducing non‐selective drug accumulation, significantly lowering therapeutic threshold doses, minimizing side effects, and improving treatment efficacy [33, 40]. However, targeting organelles remains a significant challenge owing to the complexity of physiological and biological barriers.

**SCHEME 1:**
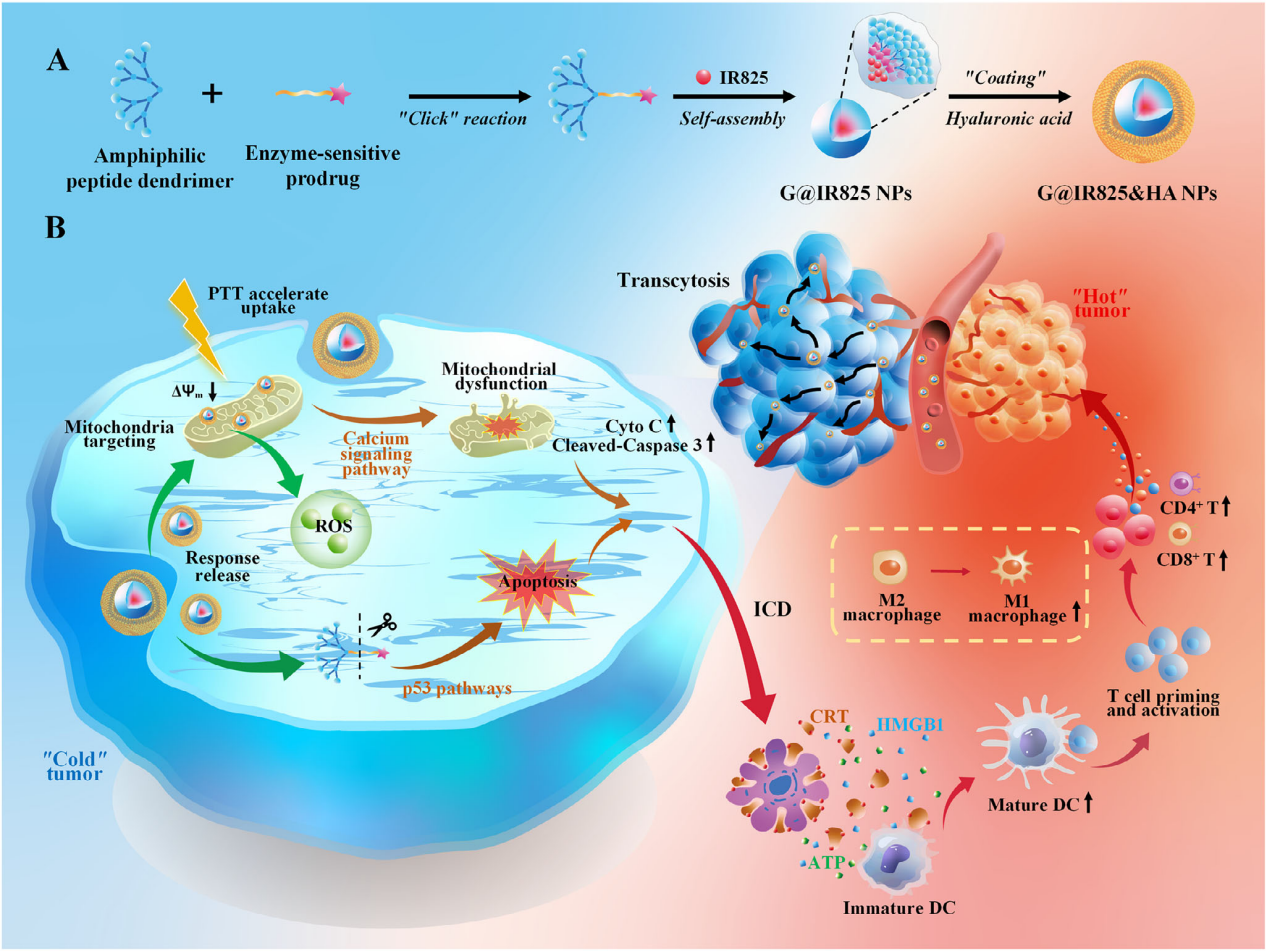
Schematic illustration of the amphiphilic peptide dendrimers‐based nanoplatform for mitochondrial targeting and synergistic chemo/photothermal therapy–induced ICD activation.

In this study, we constructed an amphiphilic peptide dendrimer‐based drug delivery system for a combined tumor therapy through targeting mitochondria and activating ICD in tumorous cells. As illustrated in Scheme 1, the amphiphilic peptide dendrimer was used to co‐deliver photothermal and chemotherapeutic agents (Gemcitabine, Gem) via an enzyme‐sensitive covalent linker and a nanoprecipitation method, respectively. The layer‐by‐layer deposition technique was employed to fabricate hyaluronic acid (HA)‐coated nanoparticles for enhanced stability and tumor targeting. This nanoplatform was strategically engineered to address key challenges in cancer therapy. First, the nanoplatform may enhance the drug delivery efficiency by improving systemic circulation stability, promoting CD44 receptor‐mediated cellular uptake via the HA coating, and facilitating deep tumor penetration through both transcytosis and intercellular transport mechanisms. Second, by delivering the therapeutic agent to the mitochondria and generating localized hyperthermia upon NIR irradiation, the system could induce mitochondrial dysfunction and promote apoptosis, thereby achieving precise subcellular targeting and organelle‐level disruption. Third, the combination of photothermal and chemotherapeutic modalities may achieve potent direct tumor cell killing and trigger distinct ICD, thus creating a pro‐inflammatory tumor microenvironment to enhance antitumor immune responses and immunologically reprogram “cold” tumors into “hot” ones. The promising strategy by integrating targeted delivery, subcellular precision, and multi‐modal treatment into a single nanoplatform could overcome critical barriers in cancer therapy and enhance therapeutic outcomes through spatiotemporally controlled drug action and immune modulation.

## 2 Results and Discussion

### 2.1 Preparation and Characterization of G@IR825&HA NPs

The synthesis routes for two constituent molecules, G3K‐GFLG‐Gem and IR825, are presented in Figures S1 and S2. The chemical structures of two molecules were confirmed by proton nuclear magnetic resonance (^1^H NMR), mass spectrum (MS), and ultraviolet‐visible spectra (UV–vis), confirming successful synthesis of G3K‐GFLG‐Gem and IR825 (see Section S1.2 and Figures S3–S6). G@IR825&HA nanoparticles (NPs) were fabricated via a two‐step process. Hydrophobic IR825 were initially encapsulated into the core of G3K‐GFLG‐Gem to form G@IR825 via a nano‐precipitation method. Subsequently, hyaluronic acid was coated onto the surface of G@IR825 via electrostatic adsorption, forming G@IR825&HA nanoparticles.

**FIGURE 1:**
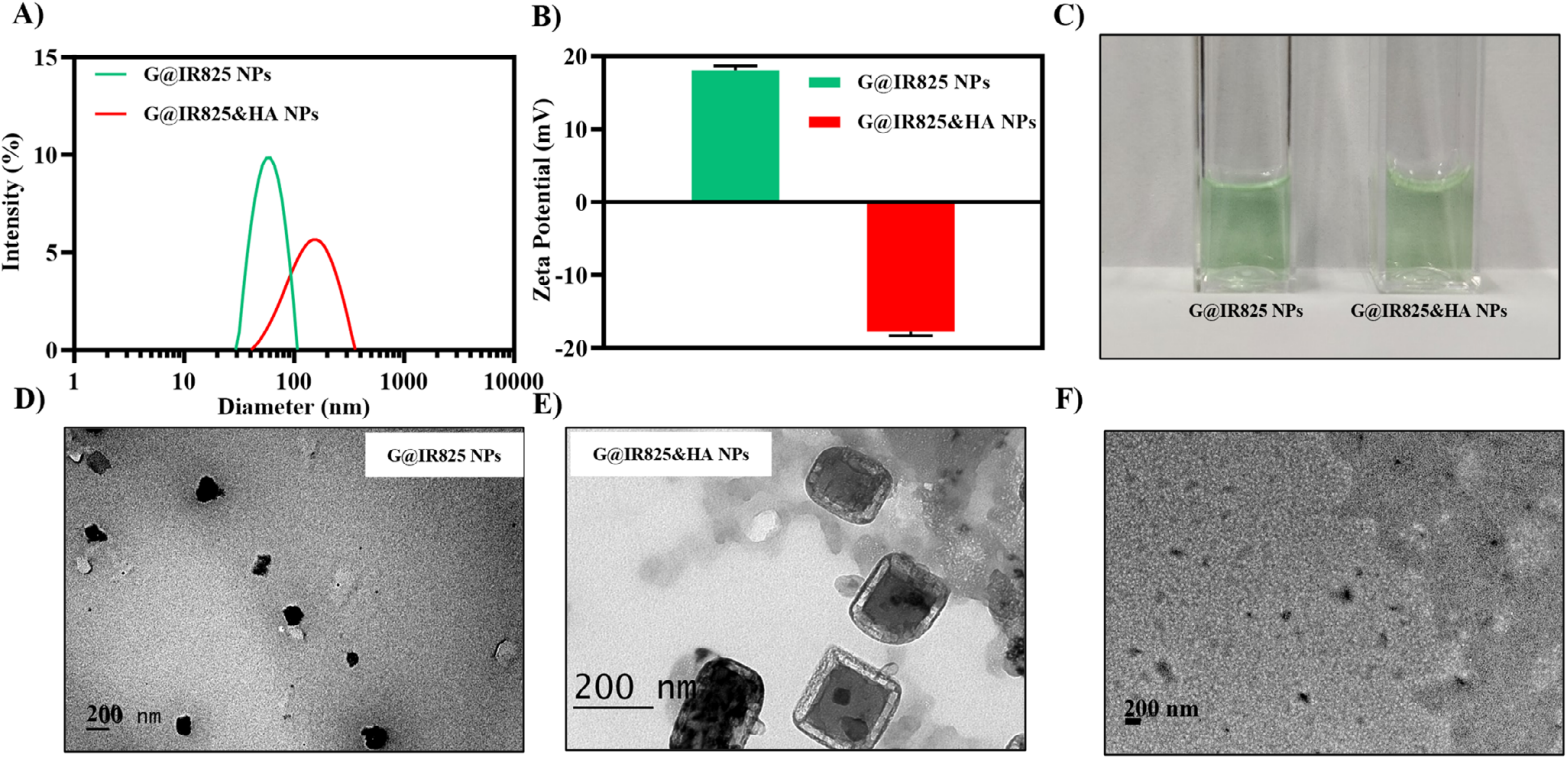
Physicochemical properties of G@IR825&HA NPs. (A–C) Particle sizes, zeta potentials and photograh of G@IR825 and G@IR825&HA NPs in ultrapure water. (D,E) TEM images of G@IR825 NPs and G@IR825&HA NPs. Scale bar: 200 nm. (F) TEM images of G@IR825&HA NPs after incubation with an enzyme mixture (papain and HAase). Scale bar: 200 nm.

As shown in Figure 1A,B, the G@IR825 NPs exhibit a particle size of 41.7 ± 1.1 nm with a positively charged surface potential of +15 ± 0.6 mV. After HA coating, the G@IR825&HA NPs size increases to 186.77 ± 22 nm, while the surface potential changes from positive to negative (−17.7 ± 0.5 mV). Both G@IR825 and G@IR825&HA nanoparticles display a light green and transparent appearance without visible aggregation or precipitation (Figure 1C). As shown in Figure 1D,E and Figure S7, transmission electron microscopy (TEM) analysis reveals that G@IR825 NPs display a uniform spherical morphology, whereas G@IR825@HA NPs exhibit a rod‐like morphology, indicating successful formation of an HA coating around the core nanoparticles. Additionally, FT‐IR spectra (Figure S8) support attachment of the outmost HA layer to the nanoparticles.

To evaluate physiological degradation of the G@IR825@HA NPs, they were incubated with an enzyme mixture containing papain and hyaluronidase (HAase) at 37°C for 24 h, mimicking the enzymatic activity in a tumor microenvironment. As shown in Figure 1F, TEM images reveal a pronounced reduction in the nanoparticle size after enzymatic treatment, supporting the degradation of the hyaluronic acid layer. This size reduction may enhance nanoparticle penetration through the dense extracellular matrix of tumors, thereby facilitating deep drug delivery and improving the therapeutic efficacy [1, 41].

### 2.2 Photothermal Conversion Performance of G@IR825&HA

**FIGURE 2:**
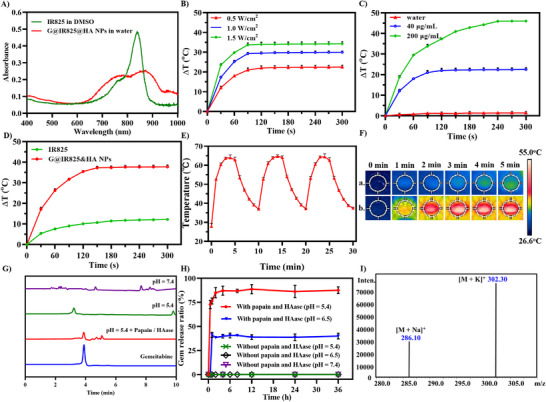
Characterizations of G@IR825&HA NPs in vitro. (A) UV–vis spectra of NPs and IR825 solutions. (B) Heating curves of G@IR825&HA NPs at the same concentration under laser irradiation at different powers (0.5, 1.0, and 1.5 W/cm^2^). (C) Heating curves of NPs solutions at different concentrations of IR825 (40 and 200 µg/mL) under irradiation, while pure water as control. (D) Heating curves of NPs and IR825 at the same concentration of IR825. (E) Photothermal stability of NPs solutions under three on‐off cycles of irradiation. (F) Infrared thermogram of (a.) pure water and (b.) NPs. Enzyme‐responsive drug release behavior of G@IR825&HA NPs in vitro was evaluated through (G) HPLC profiles of the Gemcitabine (Gem) solutions and G@IR825&HA NPs incubated with or without papain/HAase (pH = 7.4 or 5.4) and (H) cumulated release curves of Gem from G@IR825&HA NPs in PBS with or without papain/HAase (pH = 7.4, 6.5 or 5.4) (*n* = 3). (I) ESI‐MS of the released sample.

Photothermal agents should exhibit outstanding photothermal performance, efficiently absorbing and converting light energy into heat upon irradiation. We assessed the in vitro photothermal property of G@IR825&HA NPs. Compared with IR825 in DMSO, the UV absorption spectrum of NPs in water is broadened and the absorbance is slightly reduced (Figure 2A), which may be attributed to the intermolecular interaction of IR825 in the hydrophobic core of NPs.

For all experiments involving laser irradiation, “+ L” indicates exposure to a laser (808 nm, 1.0 W/cm^2^, 5 min), unless otherwise specified. As depicted in Figure S9A,B and Figure 2B,C, both IR825 and G@IR825&HA NPs exhibit a rapid temperature increase under laser irradiation, and the temperature rise is agent dose‐ and laser power‐dependent. Surprisingly, at an equivalent IR825 concentration, G@IR825&HA NPs exhibit a superior photothermal conversion efficiency (Figure 2D). Moreover, temperature rise of G@IR825&HA NPs remains steady after three on‐off cycles, whereas the free IR825 exhibits a notable decline in the temperature rise (Figure 2E; Figure S9C). The photothermal conversion efficiency (η) of G@IR825&HA NPs (15.27%) is higher than free IR825 (10.6%) as shown in Figure S9D,E. These may be attributed to the unique properties of amphiphilic dendrimers, which enhance light absorption, protect against photobleaching and thermal degradation, and reduce energy dissipation for efficient heat conversion [42]. Infrared thermographic images (Figure 2F) confirm that the nanoparticle group exhibits a rapid and sustained temperature increase upon laser irradiation, and the temperature reaches above 50°C within 3 min and continues to rise over the 5‐min exposure period. These findings validate an efficient photothermal conversion performance and high stability of G@IR825&HA NPs in vitro, supporting their application in photothermal therapy in vivo.

### 2.3 Enzyme‐Sensitive Drug Release from G@IR825&HA

To verify the enzyme‐responsive drug release behavior of G@IR825&HA NPs, papain was selected to simulate the enzyme in the tumor environment since it has similar in vitro catalytic activity to that of cathepsin B overexpressed in tumor cells [43, 44]. As shown in Figure 2G,H, a burst drug release (∼80% in 3 h) is observed after incubation with papain and HAase (pH 5.4). Rapid drug release may be attributed to enzymatic cleavage of the enzyme‐sensitive peptide (GFLG) linker by papain, which leads to destabilization of the nanostructure and acceleration of the liberation of conjugated gemcitabine. In addition, HAase‐mediated degradation of the HA shell may facilitate enzyme accessibility and promote the disassembly of the nanoparticle, collectively leading to enzyme‐triggered burst release under acidic condition. In contrast, negligible drug released is detected in 36 h for the groups without papain (pH 7.4 or 5.4). Meanwhile, the sample components with the same relative retention time (t*_R_*) as the reference substance gemcitabine (Gem) were collected and further characterized by electrospray ionization mass spectrometry (ESI‐MS) (Figure 2I). The presence of a characteristic molecular ion peak suggests that the chemotherapeutic agent gemcitabine retains its structural integrity. The above experiments sufficiently confirmed the excellent enzyme‐sensitive drug release capacity of NPs, enabling the rapid release of active Gem, which facilitates potent and efficient cancer cell elimination.

### 2.4 Cellular Uptake and Endocytosis Pathways of G@IR825&HA

**FIGURE 3:**
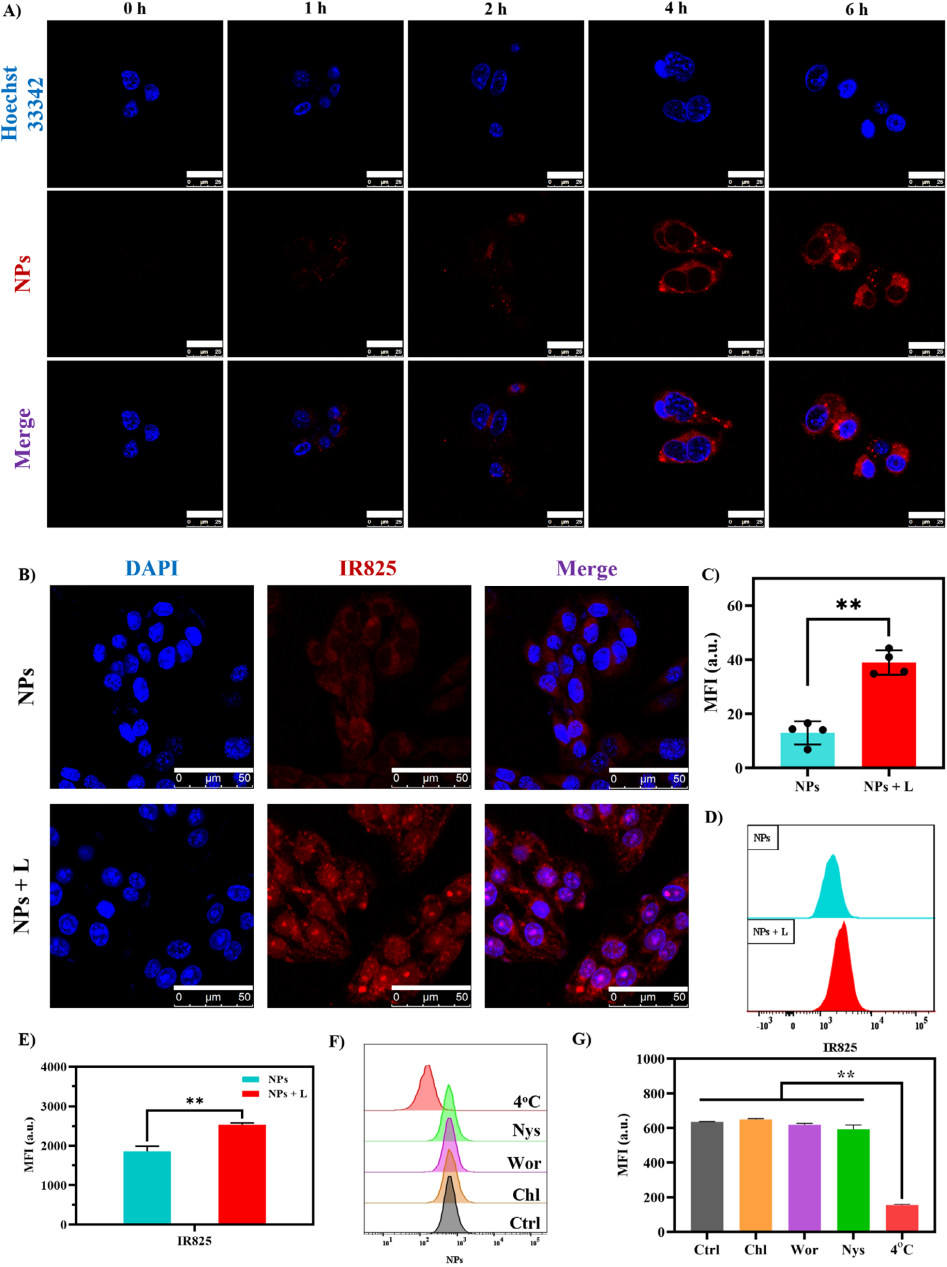
Cellular uptake and mechanisms for cellular endocytosis of G@IR825&HA NPs. (A) CLSM images of cells incubated with G@IR825&HA NPs for 0, 1, 2, 4, and 6 h. Blue for the nucleus stained by Hoechst33342. Red for IR825 in G@IR825&HA NPs. Scale bar: 25 µm; (B) CLSM images of cells treated with Cy5‐labeled G@IR825&HA NPs for 4 h under laser irradiation and (C) semi‐quantitative analysis of CLSM images. Blue for the stained nuclear; Red: for IR825 signal. Scale bar: 50 µm. (D) Flow cytometry for NPs uptake after PTT activation and (E) quantitative analysis of the IR825 fluorescence intensity. (F) Flow cytometry and (G) quantitative mean fluorescence intensity (MFI) to evaluate the impact of different endocytosis inhibitors and low‐temperature (4°C) on cellular uptake of NPs to verify the endocytosis pathway of the NPs. Nys: Nystatin; Wor: wortmannin; Chl: Chlorpromazine; and Ctrl: control without pretreatment. Unless otherwise specified, data throughout this study are presented as the mean ± SD (^*^ *p* < 0.05, ^**^ *p* < 0.01, and ^***^ *p* < 0.001).

To assess the cellular uptake efficiency of G@IR825&HA NPs, 4T1 cells were examined via confocal laser microscopy to monitor nanoparticle internalization at 1, 2, 4, and 6 h post incubation. The 4T1 cell line (murine breast cancer cell) was purchased from the National Collection of Authenticated Cell Cultures (Shanghai, China; Catalog No. SCSP‐5056, Order No. 261010, placed on August 14, 2023, and received on August 23, 2023); and was confirmed to be free of mycoplasma contamination. Unless otherwise stated, all experiments were conducted using 4T1 cells incubated with 1640 containing 10% (v/v) FBS (Umedium, He Fei, China, see Supporting Information). The fluorescence intensity of IR825 increases with an extension of the incubation time (Figure 3A), indicating a time‐dependent enhancement in cellular uptake of G@IR825&HA NPs. After a 4‐h incubation, a notably high uptake efficiency is observed. Additionally, a comparable time‐dependent uptake trend is observed for G@IR825 NPs, although with relatively lower fluorescence intensity compared to G@IR825&HA NPs (Figure S10). This result suggests that HA modification may contribute to enhanced cellular uptake.

Subsequently, cellular uptake of G@IR825&HA NPs with or without laser irradiation was evaluated via confocal microscopy. As illustrated in Figure 3B,C, cells subjected to NIR laser irradiation display noticeably enhanced red fluorescence signals compared to the non‐irradiated group, suggesting that activation of the photothermal effect facilitates NP internalization. Notably, laser‐irradiated cells exhibit a more widespread intracellular fluorescence distribution, with increased fluorescence signals observed in the perinuclear and nuclear regions, which is consistent with enhanced intracellular dispersion following increased nanoparticle internalization. Flow cytometry analysis further validates these observations (Figure 3D,E), which shows a consistent increase in the uptake efficiency upon laser irradiation. These results suggest that mild heating induced by PTT can enhance cellular internalization of the therapeutic payload‐encapsulated nanoparticles. The enhancement may be attributed to a temperature increase induced by hyperthermia, which could increase the cell membrane permeability to facilitate interactions between nanoparticles and cell membranes, thereby promoting efficient cellular internalization of nanoparticles [45].

The uptake pathways of G@IR825&HA NPs were determined via flow cytometry. Figure 3E,F shows that internalization inhibitors such as wortmannin and cytochalasin do not affect the efficiency of internalizing G@IR825&HA NPs, whereas nystatin partially inhibits the internalization process. Additionally, incubation at 4°C results in undetectable nanoparticle uptake by 4T1 cells. These findings suggest that G@IR825&HA NPs are primarily internalized through an energy‐dependent mechanism, partially through a clathrin‐mediated endocytosis pathway [46].

### 2.5 Active Targeting of G@IR825&HA

**FIGURE 4:**
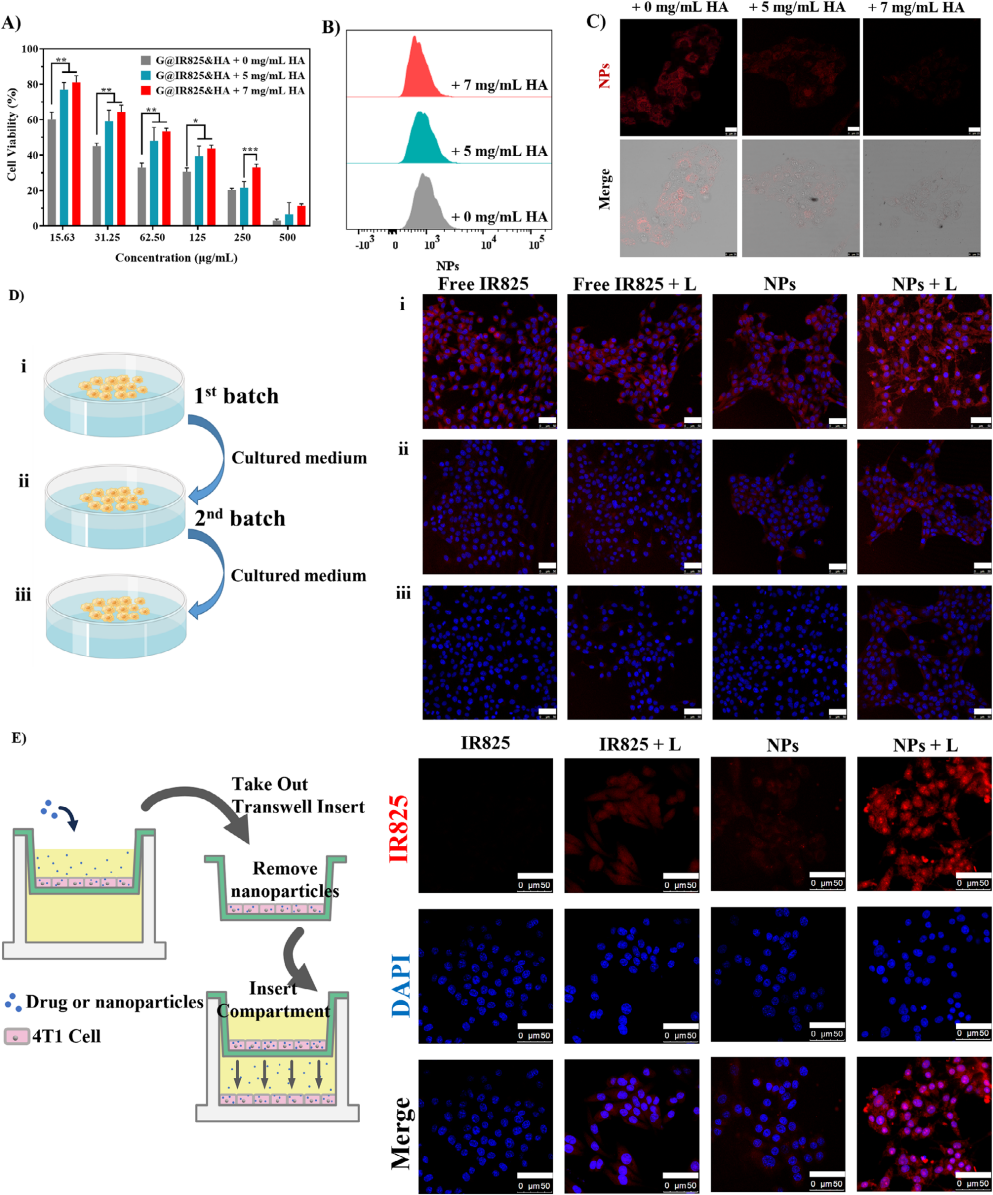
Active targeting of G@IR825&HA NPs via in vitro competitive inhibition assays. (A) Cytotoxicity of cells via the MTT assay after they were preincubated with HA at different concentrations (0, 5, and 7 mg/mL) for 4 h and treated with the NPs for 24 h. (B,C) Flow cytometry profiles and CLSM images of intracellular NPs fluorescence after the cells were preincubated with HA at different concentrations and then treated with the NPs for 4 h. Scale bar: 25 µm. (D) Intercellular transport of G@IR825&HA NPs between cells. The cells (the first batch) were cultured with the NPs at an IR825 equivalent dose of 30 µg/mL for 4 h and treated with laser irradiation. They were washed with PBS and imaged (i); These cells were cultured in fresh medium for 12 h, and the conditional medium was harvested to incubate the second batch of cells for 12 h. The second batch cells were washed and imaged (ii). The same procedures were implemented for the next couples of rounds (iii). Scale bar: 50 µm. (E) Transcytosis evaluation of G@IR825&HA NPs using a transwell co‐culture model. After laser treatment, lower‐chamber cells into which nanoparticles transported from the upper‐chamber were internalized were visualized under the CLSM. Scale bar: 50 µm.

Successful coating of a distinct HA shell on the NPs surface is observed under TEM, and the NPs may actively target the CD44 receptor on the membrane of tumor cells [47, 48]. The active targetability was assessed via an in vitro competitive inhibition experiment. As shown in Figure 4C, cells incubated with G@IR825&HA NPs exhibit strong red fluorescence signal, while cells pre‐treated with HA display a substantial reduced red fluorescence intensity, suggesting a significant reduction in cellular uptake of G@IR825&HA NPs. Furthermore, as the increase of pre‐treatment HA concentration, the red fluorescence intensity progressively decreased, which is further corroborated by quantitative flow cytometry analysis (Figure 4B; Figure S11). These findings indicate that the CD44 receptors on 4T1 cells are effectively blocked by free HA, preventing HA‐modified G@IR825&HA NPs from entering the cells through the CD44 receptor‐mediated pathway. Moreover, as shown in Figure 4A, when the concentration of a free HA solution is increased from 0 to 7 mg/mL, a progressive decrease in nanoparticle cytotoxicity is observed and the IC50 value rises from 22.54 to 70.64 µg/mL (see Table S1). These findings confirm HA coating can enhance targeted nanoparticle delivery via CD44 receptor‐mediated uptake, which could not only reduce off‐target effects but also improve cellular internalization, ultimately contributing to effective and safe cancer therapy.

### 2.6 Transcytosis of G@IR825&HA

We investigated intercellular drug transport behavior of G@IR825&HA NPs via the “infection” assay [49]. As shown in Figure 4D, obvious red fluorescence signals are detected in the cells from each batch in the NPs groups with or without laser irradiation. This result indicates that the engineered NPs can efficiently mediate drug transport between tumor cells via an endocytosis‐exocytosis process, suggesting the drug in the NPs may achieve deep tumor penetration via transcytosis. In contrast, red fluorescence signals decrease rapidly in the third batch of cells in the IR825 groups with or without laser irradiation, suggesting free IR825 has a lower capacity for intercellular transport. These results support that the active transcytosis is stemmed from rational design of the NPs. In an enzymatically rich tumor environment, the HA shell is rapidly hydrolyzed, leading to a reduction in the particle size and partial exposure of weakly positive charges (Figure S12), which may together promote efficient endocytosis and subsequent intercellular transport of drugs in the NPs [46, 49]. Interestingly, compared to the NPs group without laser irradiation, the red fluorescence intensity is stronger in the NPs + L group per batch, suggesting that photothermal therapy accelerated the internalization of the NPs, thereby facilitating intercellular transport.

To validate the transcytosis capacity of the nanoparticles, we employed a transwell‐based co‐culture system to simulate intercellular drug transport in vitro (Figure 4E). 4T1 cells incubated with nanoparticles were seeded in the upper chamber, while naive 4T1 cells were cultured on coverslips in the lower chamber. After co‐incubation, very few red fluorescence signals are detected in the cells in the lower chamber of the free IR825 group, indicating limited intercellular transport. In contrast, moderate fluorescence signals are observed in both the IR825 + L and NPs groups, suggesting that laser irradiation facilitates IR825 uptake and intercellular transfer, while encapsulation of IR825 into NPs modestly enhances its intercellular mobility. Notably, the strongest red fluorescence signals are recorded in the NPs + L group. This enhancement may arise from the rationally designed nanoparticle system and photothermal activation, both of which synertistically promote efficient cellular uptake and intercellular transport of the therapeutic payload.

Together, these results provide evidence that G@IR825&HA NPs possess an outstanding transcytosis capability. Because of efficient intercellular drug transport, this nanoplatform may overcome the poor permeability of solid tumors, thereby promoting deep drug penetration and improving therapeutic efficacy.

### 2.7 Penetration of G@IR825&HA in 3D Multicellular Tumor Spheroids (MTSs)

**FIGURE 5:**
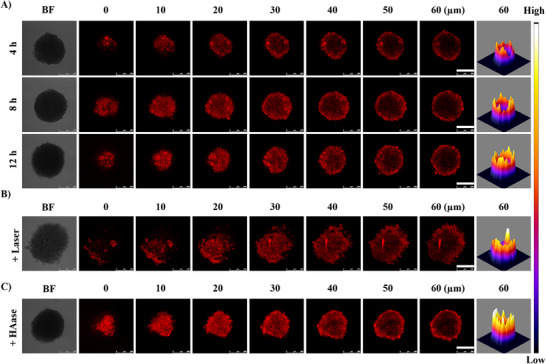
Deep penetration of G@IR825&HA NPs in multicellular spheroids (MCS) formed from cells. (A) CLSM images of MCSs after treated with NPs at different time points (4, 6, and 8 h). (B) CLSM images of MCSs after 4 h incubation with NPs, laser irradiation, and another 12 h incubation. (C) CLSM images of MCSs after they were preincubated with HAase for 4 h, treated with NPs and incubated for 12 h. Scale bar: 250 µm.

Tumor penetration of G@IR825&HA NPs into multicellular tumor spheroids (MTSs) was assessed [50]. MTSs were co‐cultured with G@IR825&HA NPs for 4, 8, and 12 h and observed under confocal laser microscopy. As depicted in Figure 5A, the penetration depth of G@IR825&HA NPs into MTSs is increased as the incubation time increased. Moreover, the results are aligned well with the intercellular drug transport results (Figure 4D,E), supporting the NPs can enhance intra‐tumoral drug distribution.

It has been demonstrated that photothermal activation facilitates the internalization of G@IR825&HA NPs into 4T1 cells (Figure 4D), and we further assessed the impact of photothermal activation on deep penetration of G@IR825&HA NPs into MTSs. As shown in Figure 5B, the fluorescence intensity in the outer layer of MTSs is significantly enhanced after laser irradiation compared to non‐irradiated MTSs, suggesting that the photothermal effect generated upon laser irradiation results in a rise in the temperature of the cells in the outer layer and an increase in the cell membrane permeability, thus promoting the uptake of the NPs by the cells in the outer layer. In addition, as shown in Figure 5C, pretreatment of tumor spheroids with hyaluronidase (HAase) markedly enhances NP penetration compared with the untreated group. The improvement in NP penetration may result from enzymatic degradation of the HA shell, which results in a smaller particle size (Figure S12) and an increase in the NP transport through the dense tumor matrix.

### 2.8 Mitochondria‐Specific Targeting and Mitochondrial Damage by G@IR825&HA

**FIGURE 6:**
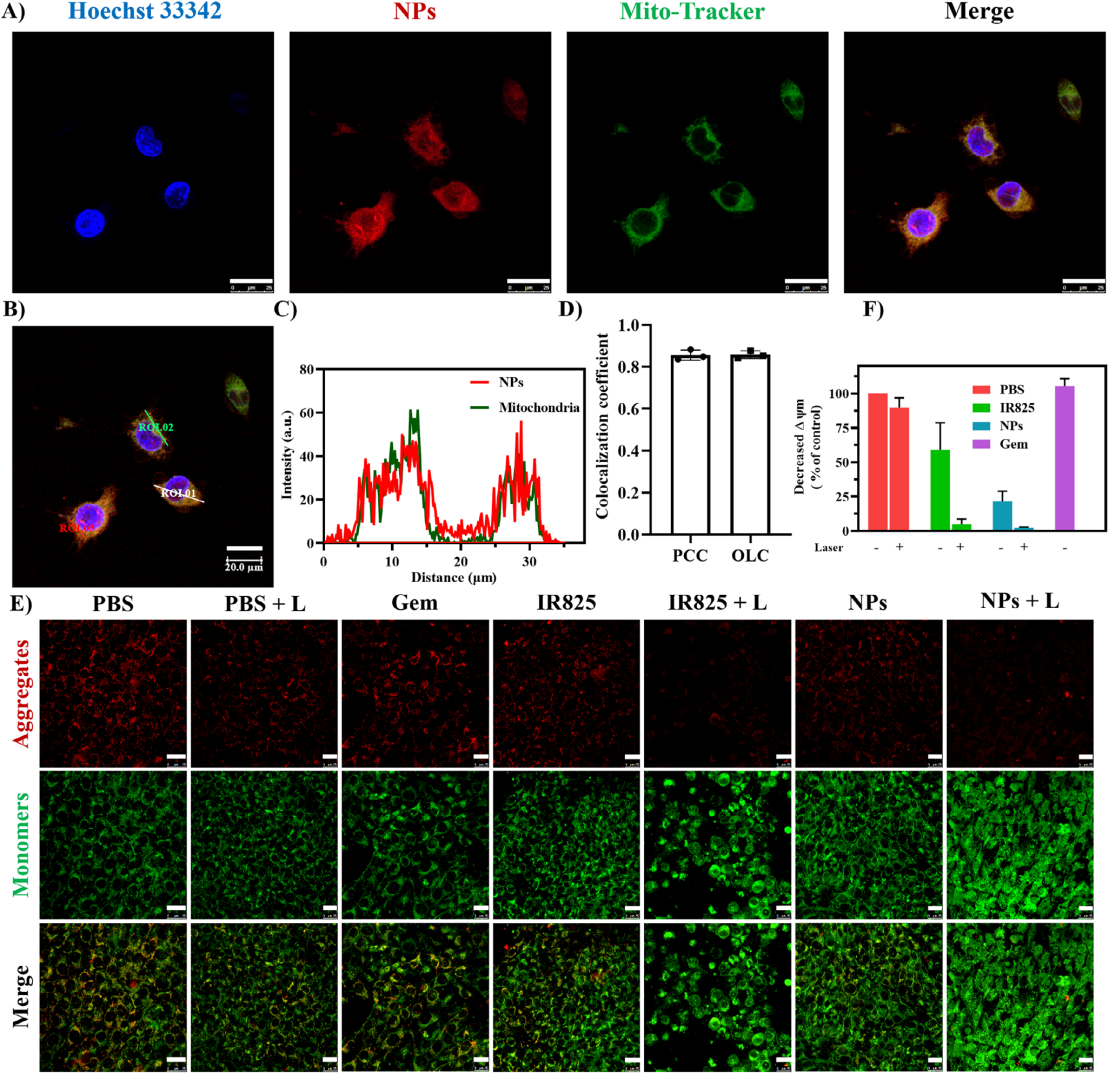
Mitochondrial targeting as well as mitochondrial damage of G@IR825&HA NPs. (A) Colocalization analysis of NPs and the mitochondria. CLSM images of cells were incubated with NPs for 4 h, followed by Mito‐Tracker staining (blue: nuclei; Red: NPs; Green: cell mitochondria). Scale bar: 25 µm. (B‐C) Frequency curves of fluorescence signal distribution at different distances obtained from co‐localization analysis (ROI for the region of interest, and three randomly selected regions for analysis: ROI 01, ROI 02, and ROI 03). (D) Pearson co‐localization coefficients as well as superposition coefficients from co‐localization analysis using Image J (*n* = 3). (E) CLSM images of cells stained with JC‐1 (2 µm) after different treatments. After staining cells, red for an aggregate form (polymer) of JC‐1, indicating intact ΔΨ_m_; and green for a monomeric form of JC‐1, indicating dissipation of ΔΨ_m_. Scale bar: 25 µm. (F) Changes in the mitochondrial membrane potential (ΔΨ_m_) indicated by the relative fluorescent intensity of JC‐1 aggregates over that of JC‐1 monomers in cells after different treatments.

Given the efficient cellular internalization of G@IR825&HA NPs, their subcellular distribution is critical for precise tumor‐targeted therapy. To this end, organelle co‐localization experiments were performed to investigate mitochondrial localization. As illustrated in Figure 6A, the red fluorescence signals of the NPs are overlapped well with the green fluorescence signals of Mito‐Tracker to yield a bright yellow signal, indicating a high level of co‐localization between the NPs and the mitochondria. Analysis of the merged confocal images via ROI line profiling (Figure 6B,C) reveals that in a randomly selected region, such as ROI 01 or ROI 02, the fluorescence signals of the NPs share a similar profile as the mitochondrial fluorescence signal along the selected line, which is consistent with visual observations. Quantitative analysis indicates the Pearson correlation coefficient (PCC) and the overlap coefficient (OLC) are above 0.8 (Figure 6D), confirming a high level of co‐localization between the NPs and the mitochondria and a high degree of mitochondrial targeting. Collectively, these results confirm that G@IR825&HA NPs preferentially accumulate in the mitochondria, providing a mechanistic basis for subsequent studies on mitochondrial disruption and stress induction.

Preferential mitochondrial accumulation of G@IR825&HA NPs can be ascribed to combined contributions of the encapsulated IR825 and physicochemical characteristics of the dendritic carrier. IR825 belongs to the heptamethine cyanine dye family, and it has been widely reported to exhibit mitochondrial affinity. Its extended π‐conjugated structure, pronounced hydrophobicity, and a delocalized cationic nature collectively contribute to mitochondrial accumulation driven by its interaction with a highly negative mitochondrial membrane potential (ΔΨm). IR825 has been reported as a mitochondria‐targeting molecule in multiple studies, supporting the intrinsic mitochondrial affinity of this fluorophore. In addition, the third‐generation lysine‐based dendrimer used in this work possesses multiple terminally exposed primary amine groups, conferring a multivalent cationic surface after cellular internalization. Previous studies have shown that cationic lipid‐like or dendritic macromolecules may exhibit a tendency to associate with mitochondria through electrostatic interactions with the negatively charged mitochondrial membranes under certain intracellular conditions. Although such interactions do not constitute organelle‐specific targeting, they may facilitate mitochondrial proximity or retention of internalized nanoparticles. Therefore, the observed mitochondrial localization of G@IR825&HA NPs is likely the result of a synergistic effect between the mitochondria‐affinitive IR825 payload and the cationic dendritic scaffold, which together promote preferential mitochondrial accumulation, as evidenced by the high co‐localization metrics observed in Figure 6A–D.

Given the observed mitochondrial targeting of NPs, we further investigated their impact on mitochondrial activity, with MMP measurements serving as a key indicator of mitochondrial integrity and potential apoptotic induction. Accordingly, a JC‐1 assay was carried out to investigate the effect of G@IR825&HA NPs on mitochondrial activity. The transformation of JC‐1 from J‐aggregates (fluorescent red) to J‐monomers (fluorescent green) during different treatments was monitored to assess the level of mitochondrial depolarization. As shown in Figure 6E, compared to the bright red fluorescence indicative of healthy mitochondria in the PBS group, the NPs group displays a remarkable decrease in the red fluorescence intensity, indicating mitochondrial damage. Following laser irradiation, remarkable green fluorescence signals and weak red fluorescence signals are observed in the NPs + L group, indicating a high degree of tumor cell mitochondrial destruction. The decline in the mitochondrial membrane potential (ΔΨ_m_) is further quantified by the red/green fluorescence intensity ratio in different treatment groups [51]. The semi‐quantitative results show that the ΔΨ_m_ in the NPs + L group is 1.7% of the control group (Figure 6F). This result may be ascribed to efficient cellular uptake of NPs into tumor cells and their excellent photothermal property, ultimately resulting in mitochondrial dysfunction. Additionally, the fluorescence signal intensity in the IR825 + L group is similar to that in the NPs + L group, suggesting that IR825 can diffuse into tumor cells and generate PTT effects efficiently. These results proved that G@IR825&HA NPs based on PTT therapeutical agent has the potential of disrupting the structure of the mitochondria and/or destroying its function, providing a promising strategy for enhancing cancer treatment efficacy.

### 2.9 In Vitro Cytotoxicity of G@IR825&HA and Live/Dead Cell Staining

**FIGURE 7:**
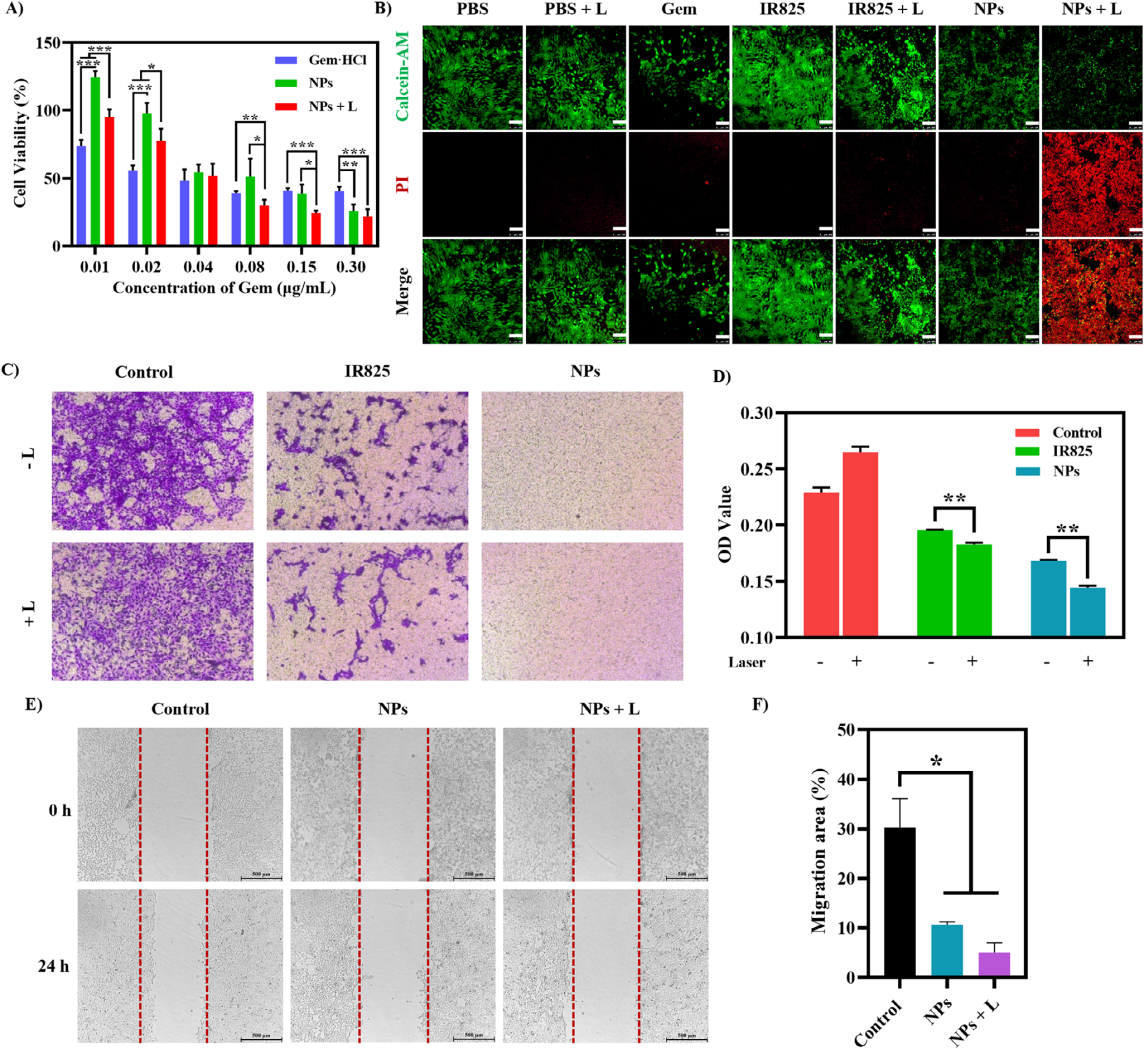
The cell inhibitory effect of G@IR825&HA NPs. (A) In vitro cell viability of NPs, NPs + L, and Gem·HCl. (B) Live/dead staining of cells after different treatments (PBS, PBS + L, IR825, IR825 + L, Gem, NPs, NPs + L) for 4 h. The live cells were stained by Calcein‐AM (green), whereas dead cells by PI (red). Scale bar: 100 µm. (C,D) Migration of cells after different treatments for 4 h in Transwell chambers (8 µm pore size) and Quantification of crystal‐violet cell staining. (E,F) Changes in the scratch area analyzed semi‐quantitatively (scale bar: 500 µm).

Mitochondrial targeting may enhance the antitumor efficacy of G@IR825&HA NPs. Their therapeutic efficacy against 4T1 cells was assessed via methyl thiazolyl tetrazolium (MTT) assays. As shown in Figure 7A, the viability of cells in NPs + L group decreased in a concentration‐dependent manner, indicating potent cytotoxicity may be induced by the combined effects of photothermal therapy (PTT) and chemotherapy. Meanwhile, as shown in Figure S13A, the free photosensitizer, IR825, exhibits a low level of cytotoxicity in the absence of irradiation, supporting its biosafety for anticancer treatment [23]. Negligible cytotoxicity toward human umbilical vein endothelial cells (HUVECs) is observed after treatment with G@IR825&HA NPs, whereas free gemcitabine induces a more pronounced reduction in the cell viability at 0.30 µg/mL (Figure S13B). These results indicate that, in the absence of laser irradiation, the nanoparticle formulation exhibits improved biocompatibility toward normal cells. To further assess cell viability, live/dead staining tests were carried out. As shown in Figure 7B, the NPs and Gem groups exhibit strong green fluorescence with minimal red signal, while intense red fluorescence signals but weak green signals are seen in NPs + L group, confirming extensive cell death in the NPs + L group. These results all indicated that an outstanding in vitro antitumor therapeutic effect.

### 2.10 Inhibition of Cell Migration

Tumor cell migration remains to be a major driver of cancer‐related mortality. Inhibition of tumor cell migration remains a substantial challenge in cancer treatment [2]. The process of tumor cell migration involves multiple critical stages, including invasion, migration, and adhesion. We conducted scratch and invasion assays to assess the impact of G@IR825&HA NPs on migration and invasion of tumor cells. In the cell scratch assay (Figure 7E,F), after 24 h of incubation, the width and the area of the scratch in the NPs + L group remain unchanged, indicating significant suppression of cell migration. In contrast, noticeable cell migration is seen in the control group and the NPs group showed a limited amount of migration. As shown in Figure 7C,D, the most significant inhibitory effect on cell invasion is found in the NPs + L group. Compared to the IR825 and NPs groups without laser irradiation, PTT significantly enhanced the inhibition of cell invasion. Therefore, rational design of the nanoplatform by leveraging the synergistic effects of chemotherapy and photothermal therapy can effectively suppress cancer cell migration and invasion. This approach holds promise in preventing tumor recurrence and metastasis in vivo [17].

### 2.11 In Vitro Immunogenic Cell Death Induction

**FIGURE 8:**
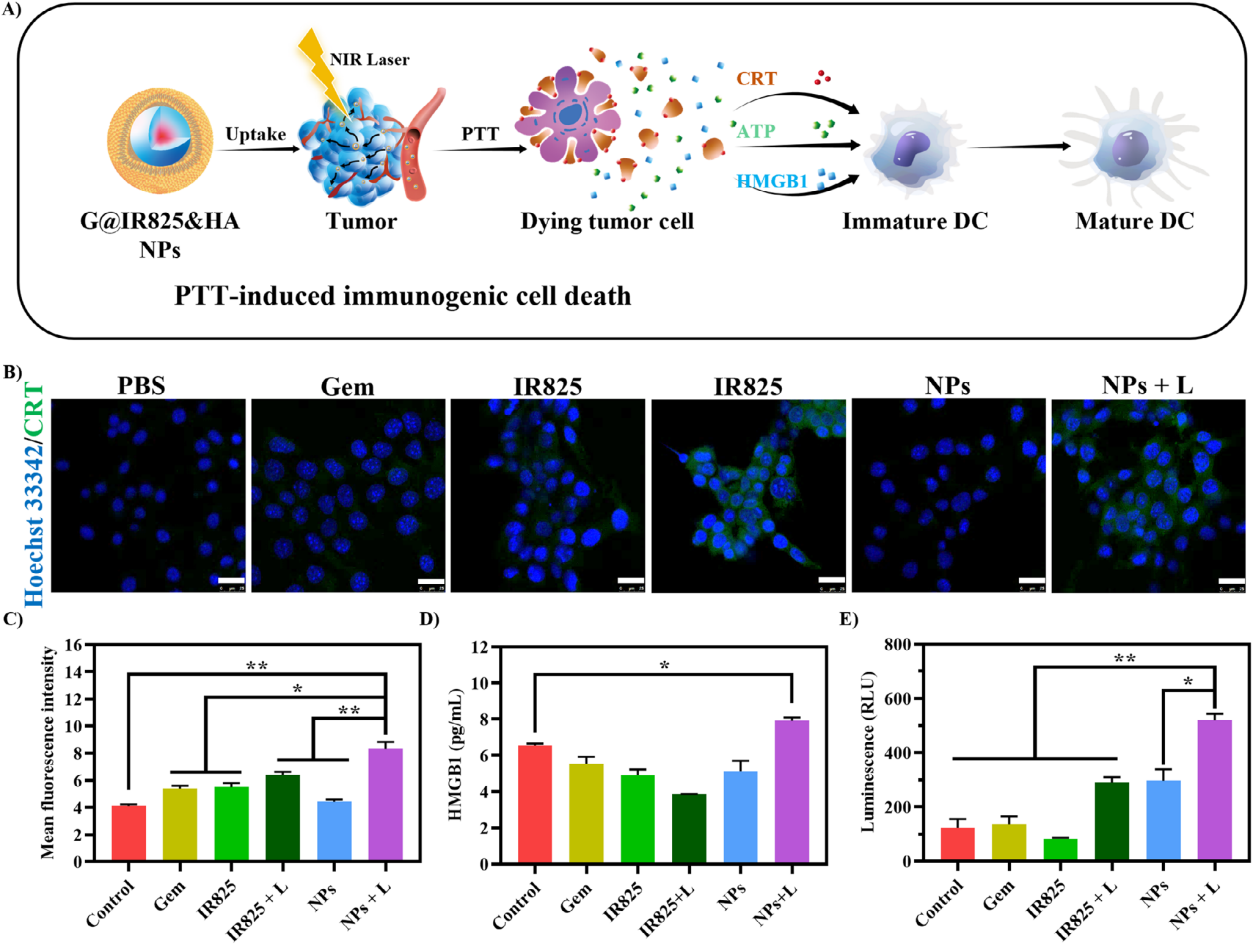
(A) Schematic illustration of PTT‐induced immunogenic cell death after cells were treated with the G@IR825&HA NPs. (B) CLSM images of CRT exposure (green fluorescence) after different treatments (Scale bar: 10 µm). (C) Semi‐quantitative analysis of the level of CRT fluorescence. (D) HMGB1 secretion levels in different treatment groups via an ELISA kit. (E) ATP secretion levels after various treatments measured via an ATP chemiluminescence kit.

To evaluate the ICD effects induced by G@IR825&HA nanoparticles, we monitored CRT exposure, HMGB1 release, and ATP secretion (Figure 8A). CRT is exposed on the surface of dying tumor cells, acting as an “eat me” signal to antigen‐presenting cells (APCs). This facilitates tumor cell phagocytosis, cytokine production, and antigen presentation, ultimately activating cytotoxic T cells and enhancing immune responses. The expression of CRT protein was detected on the membrane by immunofluorescence to evaluate the ICD‐inducing capability by monotherapy (chemotherapy or PTT) and synergistic therapy. As shown in Figure 8B, the expression of the CRT protein shown as green signals in the NPs + L group is significantly elevated compared to that in the control and NPs groups without laser irradiation, indicating that the nanoparticles under laser irradiation could induce ICD effectively due to the potent photothermal effect produced by the NPs. The HMGB1 protein can bind to receptors located on the membrane of dendritic cells (DCs), thereby initiating signaling pathways for the maturation of DCs and subsequent presentation of antigens for the activation of cytotoxic T lymphocytes (CTLs). The HMGB1 protein secreted in the cell culture media after different treatments was detected via an ELISA kit. 4T1 cells in NPs + L group display an increase in HMGB1 release (Figure 8D), confirming ICD activation. When tumor cells experience ICD, the release of intracellular ATP can be triggered, and ATP serves as a “find‐me” signal to attract immune cells for recognition. As shown in Figure 8E, substantial secretion of ATP is found when tumor cells were treated by the NPs with laser irradiation. The above results indicate that the G@IR825&HA nanoparticles can induce ICD in the 4T1 cells through photo‐immunotherapy, which is essential for the initiation of tumor immunotherapy [16].

### 2.12 Hemolysis Assay

All animal experiments have been approved by the Experimental Animal Welfare and Ethics Committee of Fujian Medical University (approval number: IACUC FJMU 2022‐0035) and conducted by following the guidelines from the Ethics Committee and National Regulations of China. To evaluate the potential of the nanoparticles for in vivo application, hemolysis assays were conducted to assess blood compatibility and biocompatibility of the nanoparticles prior to their in vivo experiments. As shown in Figure S14, incubation with G@IR825&HA NPs at a concentration of up to 500 µg/mL does not result in significant hemolysis. The hemolysis rate of the nanoparticles remains below 5%, indicating good compatibility with blood plasma. These results collectively suggest that the nanoparticles exhibit a high level of safety, supporting their potential for safe in vivo.

### 2.13 Ex Vivo Distribution Experiments of NPs

**FIGURE 9:**
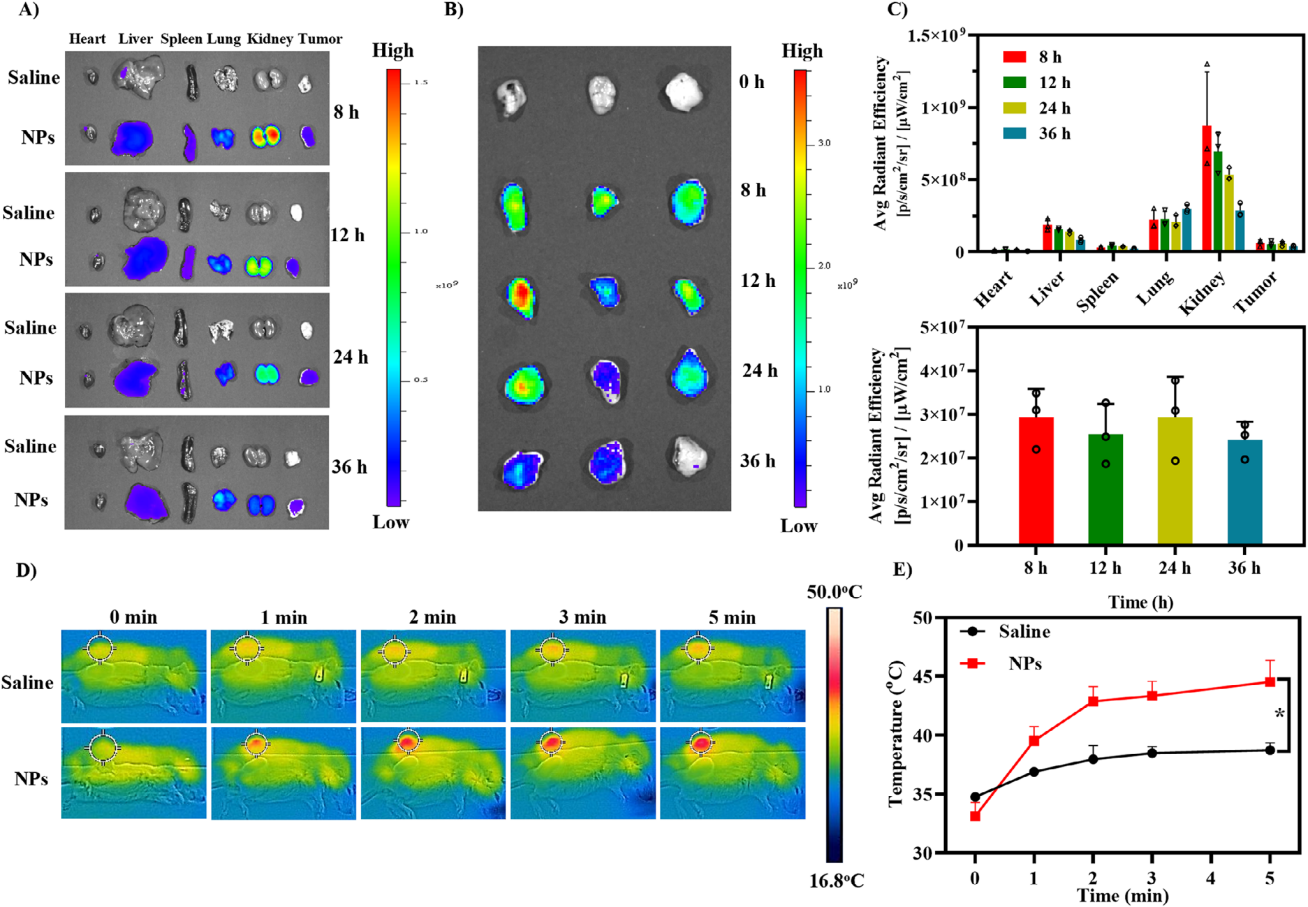
Tumor‐targeting performance of G@IR825&HA NPs. (A) Ex vivo fluorescence images of main organs and tumors collected from the 4T1 tumor‐bearing mice at a specified time point (8, 12, 24, and 36 h) after injection of saline and Cy5‐labeled G@IR825&HA NPs. (B) Ex vivo fluorescence images of tumors (*n* = 3). (C) Semi‐quantitative results for the average fluorescence intensity in major isolated organs and tumors after administration. (D) Photothermal images of G@IR825@HA NPs or Saline‐treated 4T1 tumor‐bearing mice upon irradiation at 24 h post‐injection. (E) Quantitative results for the temperature change.

To investigate the distribution of nanoparticles within a biological system, Cy5‐labeled G@IR825&HA NPs were i.v. injected into nude mice bearing tumors. Their major organs were subsequently collected for imaging. As shown in Figure 9A–C, an obvious accumulation of nanoparticles within tumors is observed at 8 h post‐injection. The fluorescence signal of the tumor region is progressively intensified over time, with a clear accumulation observed at 12 h. Notably, the tumors show strong fluorescence signals after 36 h postinjection, suggesting prolonged retention. These results indicate that the G@IR825&HA NPs can selectively accumulate at tumor sites and they can be retained for an extended period, which is crucial for enhancing therapeutic efficacy.

### 2.14 In Vivo Photothermal Effects of NPs

Remarkable tumor‐specific accumulation of G@IR825&HA NPs may result in a potent photothermal effect at the tumor site in vivo. As shown in Figure 9D,E, there is a rapid temperature elevation at the tumor site in the G@IR825&HA NPs group under laser irradiation. The temperature at the tumor site reaches 46.5°C within 3 min and it is maintained over the 5‐min irradiation period, whereas there is a slight increase in the temperature from 35.1°C to 39.8°C in the saline group. As depicted in Figure S15, a color change is observed at the tumor site in the G@IR825@HA NPs‐treated mice 2 h after laser irradiation, and an original pink color is transitioned to a dark purple color, which may be ascribed to the hyperthermia‐induced vascular rupture at the tumor site and localized congestion [52]. In contrast, the saline group upon laser irradiation showed minimal changes, confirming the photothermal effect mediated by the G@IR825&HA NPs. Therefore, these experimental results demonstrate that G@IR825@HA NPs can effectively accumulate within mouse solid tumors and exhibit excellent photothermal efficacy.

### 2.15 In Vivo Antitumor Effects of NPs

**FIGURE 10:**
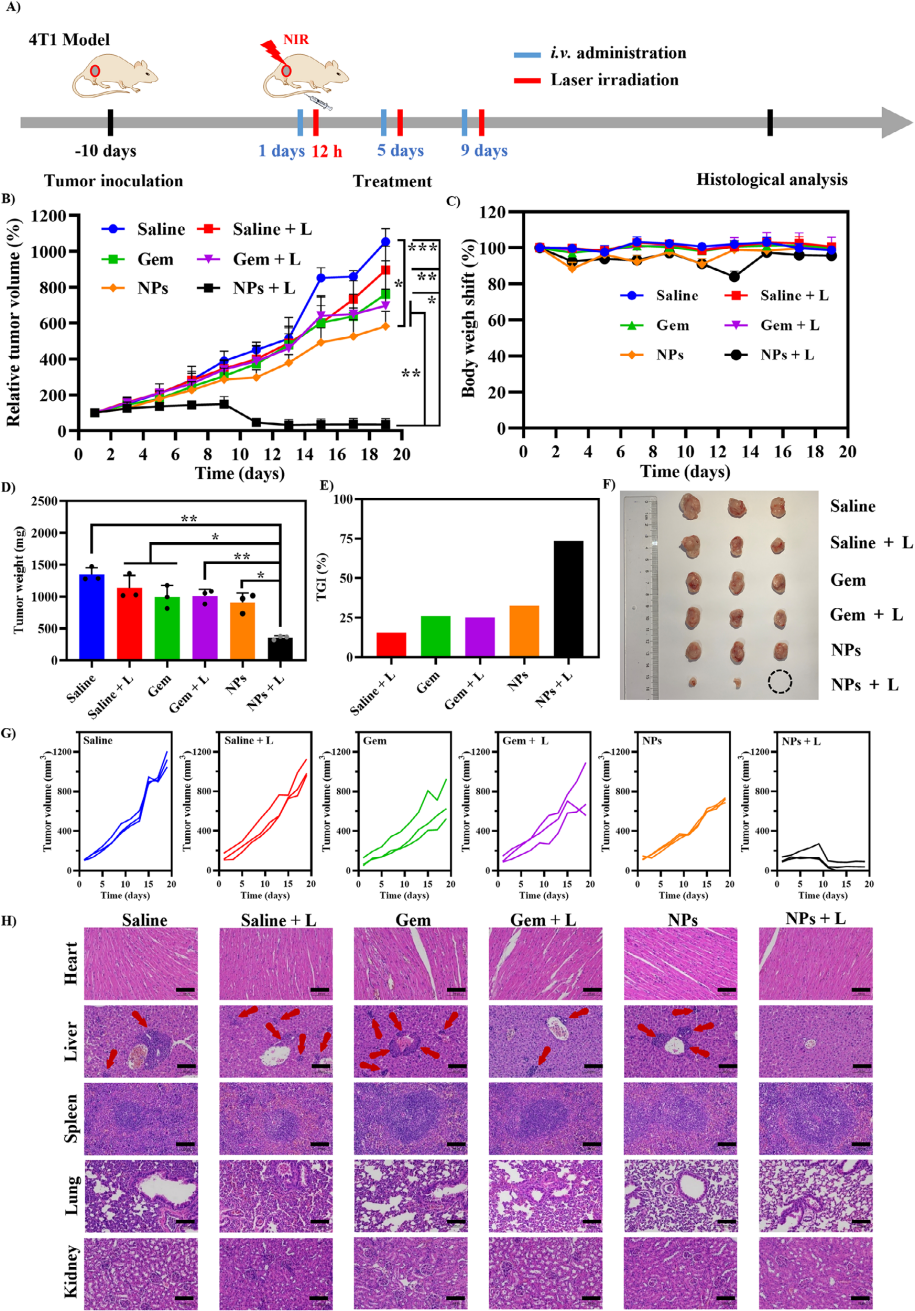
In vivo antitumor effect of G@IR825&HA NPs. (A) Treatment schedule. (B,C) The tumor growth curves expressed by the relative tumor volume and the body weight changes. (D) Weights of the tumors harvested from the sacrificed mice on the 19th day. (E) Tumor growth inhibition rates (TGI). (F) Images of tumors harvested from the sacrificed mice on the 19th day (*n* = 3). (G) Tumor growth curves of per mouse during the treatment period. (H) Hematoxylin‐eosin (HE) staining analysis of major organs of mice at the end of the treatment period.

The treatment schedule is shown in Figure 10A. As shown in Figure 10B, the tumor growth of the NPs + L group is significantly inhibited during the entire period of experiment. The NPs group without laser irradiation demonstrated superior antitumor efficacy compared to the free Gem group, which may be attributed to the enhancement in the stability of gem in bloodstream after its incorporation into nanoparticles, thereby reducing its rapid metabolism and facilitating its accumulation at the tumor site [53]. Mice were sacrificed at the end of the treatment period, and tumors were collected and photographed. As shown in Figure 10F, the tumors in the NPs + L group are significantly smaller than those in the other groups. Meanwhile, complete tumor eradication is observed in some of the mice in the NPs + L group, which supported a robust synergistic effect of our nanoparticles. Quantitative evaluation of tumor weights and tumor growth inhibition (TGI) confirms the volume reduction observations (Figure 10D,E). The tumor weight in the NPs + L group is 355.6 ± 29.7 mg (*p* < 0.001 vs the Saline group and *p* < 0.005 vs the Gem group), corresponding to a TGI of 73.6%. In contrast, the tumor weight in the Gem group is 995.5 ± 179.6 mg with a modest TGI of 26.0%. In the saline group, the tumors grow rapidly. Laser irradiation alone does not exert an inhibitory effect, with a low TGI of 15.6%.

Meanwhile, hematoxylin‐eosin staining (H&E) of major organs in the treated mice were conducted. As shown in Figure 10H, no histological abnormalities are observed in the main organs of the mice in the NPs + L group. In contrast, the other treatment groups exhibit varying levels of tumor metastasis within the liver tissues. Furthermore, the body weight of the mice in all groups (Figure 10C) remains relatively stable during the entire period of treatment, and the mice maintain normal hair and behavioral patterns, indicating excellent tolerability of the treatment with nanoparticles and laser irradiation. To further assess the biocompatibility of G@IR825&HA NPs, blood routine examination was carried out. As shown in Figure S16A–G, the hematology marker values are predominantly within the normal ranges, indicating great biocompatibility of the NPs. Collectively, these results suggest that G@IR825&HA NPs from a rational design could be used for safe and effective tumor treatment. The nanoparticle formulation improves the in vivo stability and tumor‐site accumulation of gemcitabine. By integrating hyaluronic acid‐mediated tumor targeting, nanocarrier encapsulation‐aided enhanced drug retention, and near‐infrared‐triggered photothermal effects, the system achieves synergic chemotherapy and photothermal therapy. This dual‐mode therapy enhances drug bioavailability and chemosensitivity while maintaining excellent biocompatibility, presenting a promising approach for precise and potent cancer treatment.

### 2.16 In Vivo Survival Rates

**FIGURE 11:**
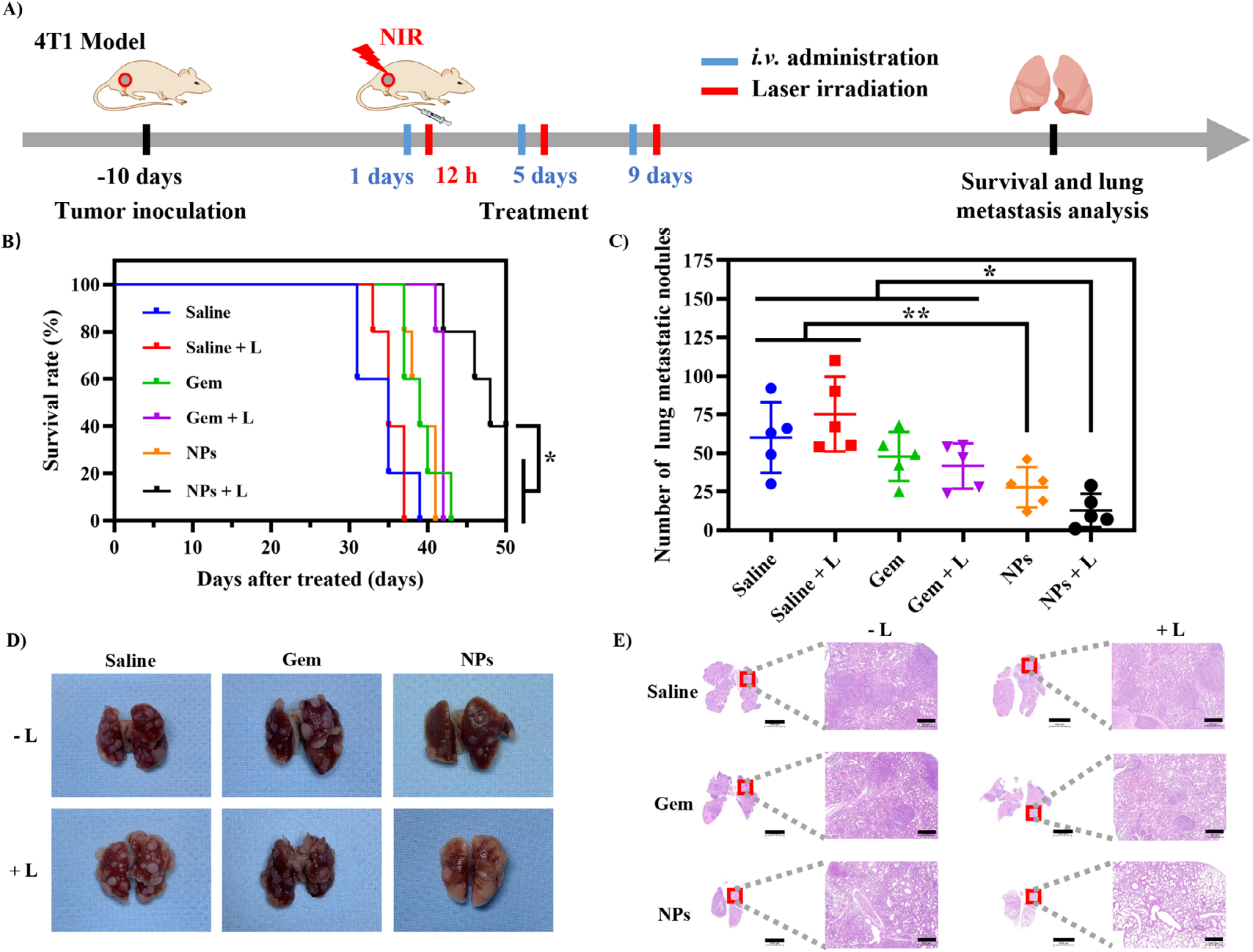
Inhibition of lung metastasis of breast cancer by NPs in vivo. (A) Treatment schedule. (B) Survival analysis curve. (C) The number of lung and lobar metastatic nodules in the harvested lung tissue (*n* = 5). During the survival experiment, when the tumor volume of tumor‐bearing mice was more than 2000 mm^3^ or the animal died naturally, the mice were sacrificed, and the lung tissue was harvested. (D) Image of the lung tissue. White dots for pulmonary metastatic nodules. (E) H&E analysis of the lung tissue.

Inspired by successful in vitro anti‐metastases and outstanding in vivo anti‐tumor effects, we evaluated the capacity of the G@IR825&HA nanoplatform in inhibiting tumor metastasis and improving the overall survival of mice. And the treatment schedule is present in Figure 11A. When the tumor volume reached 2000 mm^3^, the mice were humanely euthanized, and their lung tissues were extracted and stained with hematoxylin and eosin (H&E). As shown in Figure 11B, all mice in the saline group die within 39 days, with a median survival period of 35 days, and the group subjected to laser irradiation alone exhibits a similar survival rate benefit with a median survival of 35 days, indicating ineffectiveness of laser exposure to the mice in the absence of therapeutic agents. As observed in Figure 11C,D, the lung tissues in both groups (saline and laser irradiation alone) exhibit numerous tumor nodules, which are depicted by white dots, indicating tumor metastases after treatment with saline or laser irradiation. The corresponding H&E staining images (Figure 11E) confirm dense clusters of metastatic 4T1 tumor cells within the pulmonary parenchyma. In contrast, the mice treated with free gemcitabine (Gem) and Gem + L exhibit modest improvement in the survival, with median survival period extends to 39 and 42 days, respectively. Impressively, the NPs + L group exhibit superior therapeutic performance, achieving a 100% survival rate on day 40 and 40% survival on day 50. Compared to other groups, the NPs + L‐treated mice display a longer survival time and significantly fewer tumor nodules in their lungs.

These findings underscore the therapeutic potential of the rationally designed nanoplatform. Synergistic chemo‐photothermal therapy markedly enhances antitumor efficacy and effectively suppresses metastasis, thereby prolonging survival of the treated mice. Integrating targeted drug delivery with photothermal activation amplifies cytotoxicity of chemotherapy to tumor cells and simultaneously reshapes the tumor microenvironment to hinder metastatic progression. Such a combined approach not only achieves efficient local tumor ablation but also prevents distant recurrence, confirming that ICD‐mediated synergistic chemo‐photothermal therapy can enhance therapeutic efficacy and improve long‐term survival outcomes.

### 2.17 Transcriptomic Analysis of Synergistic Therapeutic Effects of NPs on Cellular Gene Expression

**FIGURE 12:**
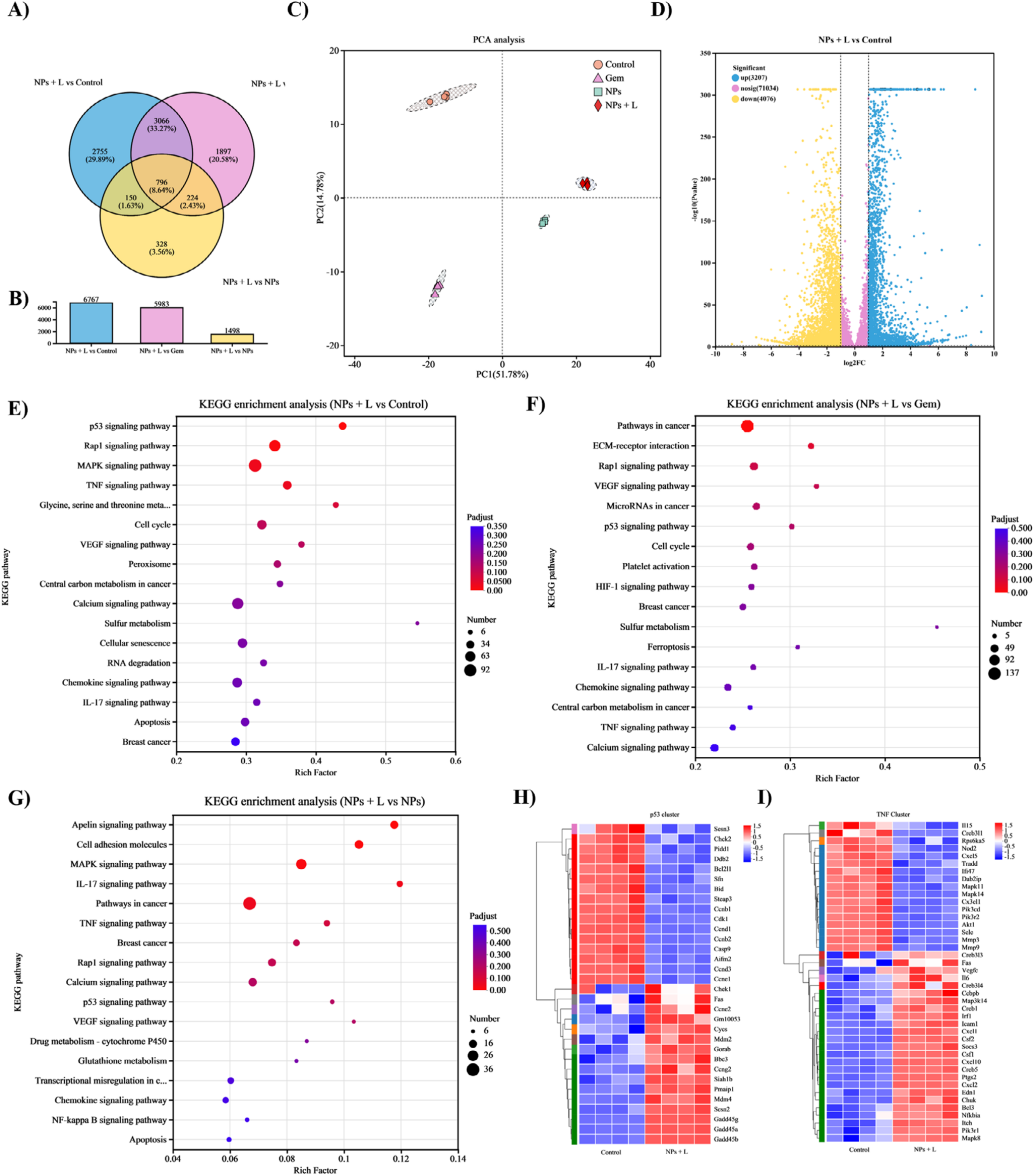
Transcriptional profiling of 4T1 cells after NPs treatment via RNA sequencing (RNA‐seq). (A) Venn diagram for DEGs to illustrate the number of genes with altered expression. (B) Statistical analysis of DEGs. (C) PCA analysis of RNA‐seq data. (D) Volcano plot for DEGs in the NPs + L group versus the control group. “No sig” for genes without significant differential expression. (E–G) KEGG pathway enrichment analysis. (H,I) Heatmaps for differentially transcribed genes in the p53‐associated and TNF‐associated clusters between the control group and the NPs + L group.

To reveal the molecular mechanisms underlying the synergistic effects of NPs, RNA sequencing was performed to compare transcriptomic changes after different treatments. Differentially expressed genes (DEGs) were identified and analyzed through Gene Ontology (GO) and Kyoto Encyclopedia of Genes and Genomes (KEGG) enrichment to determine their associated biological processes and signaling pathways. As shown in Figure 12A,B, distinct gene expression profiles are observed in different treatment groups. Compared to the control group, there are 7283 DEGs including 3207 up‐regulated DEGs and 4076 down‐regulated DEGs (Figure 12C) in the NPs + L treatment group, compared to 6440 DEGs (3119 up‐regulated and 3321 down‐regulated, Figure S17) in the Gem treatment group. Moreover, 1868 DEGs (894 up‐regulated and 974 down‐regulated, Figure S17) are identified by comparison between the NPs and NPs + L groups. These results indicate that the group treated with NPs + L displays a broad range of transcriptional changes, corroborating the strong synergistic effect of chemotherapy in combination with photothermal therapy.

To understand the biological significance of these transcriptional changes, KEGG pathway enrichment analysis was conducted for the DEGs. As shown in Figure 12F–H, genes involved in the cell cycle‐associated pathways are significantly enriched in the NPs + L group, including the p53, Rap1, MAPK, tumor necrosis factor‐α (TNF‐α), and interleukin‐17 (IL‐17) signaling pathways, compared with the control and NPs groups. Enrichment of the p53 signal suggests robust activation of DNA damage‐ and stress‐responsive checkpoints, leading to cell cycle arrest and apoptosis. In addition, pathways associated with apoptosis and breast cancer progression are highly enriched. These findings suggest that the NPs + L treatment has a great impact on cell cycle regulation and apoptosis that contribute to synergistic, efficacious therapeutic effects of the NPs in breast cancer therapy. Notably, the MAPK and Rap1 signaling pathways, which are closely associated with cellular responses to thermal stress and growth factor signaling, may be activated by mild hyperthermia induced by photothermal therapy, thereby sensitizing tumor cells to gemcitabine‐mediated cytotoxicity. Specifically, heat shock proteins generated from photothermal therapy (PTT) in combination with the chemotherapeutic action of Gem may cooperatively activate p53‐dependent apoptotic signaling, thereby promoting tumor cell death. Additionally, the enrichment of TNF and IL‐17 signaling pathways indicates reprogramming of the tumor immune microenvironment, which could help alleviate immunosuppressive signaling and enhance antitumor immunity. Notably, calcium signaling pathways are also significantly enriched, which are closely associated with the mitochondrial function and apoptotic regulation. Dysregulated calcium homeostasis may induce mitochondrial membrane depolarization, excessive reactive oxygen species production, and activation of intrinsic apoptotic pathways, leading to mitochondrial dysfunction triggered by the NPs + L treatment. Collectively, these KEGG enrichment results indicate that the synergistic therapeutic efficacy of NPs is mediated through coordinated regulation of cell cycle control, stress‐responsive signaling, apoptosis, immune‐associated pathways, and mitochondrial dysfunction.

Furthermore, principal component analysis (PCA) reveals that a distinct cluster is seen in the NPs + L samples, and the cluster is distinctly separated from those in other treatment groups, indicating a unique transcriptional state. Consistent with this observation, heatmap visualization of representative DEGs confirms up‐regulation of apoptosis‐associated genes and down‐regulation of cell cycle–associated genes in the NPs + L group. These results suggest that the synergistic therapeutic effect arises from coordinated transcriptional reprogramming, leading to disrupted tumor cell proliferation and enhanced apoptotic signaling.

### 2.18 In Vivo Immunological Cell Population Analysis

**FIGURE 13:**
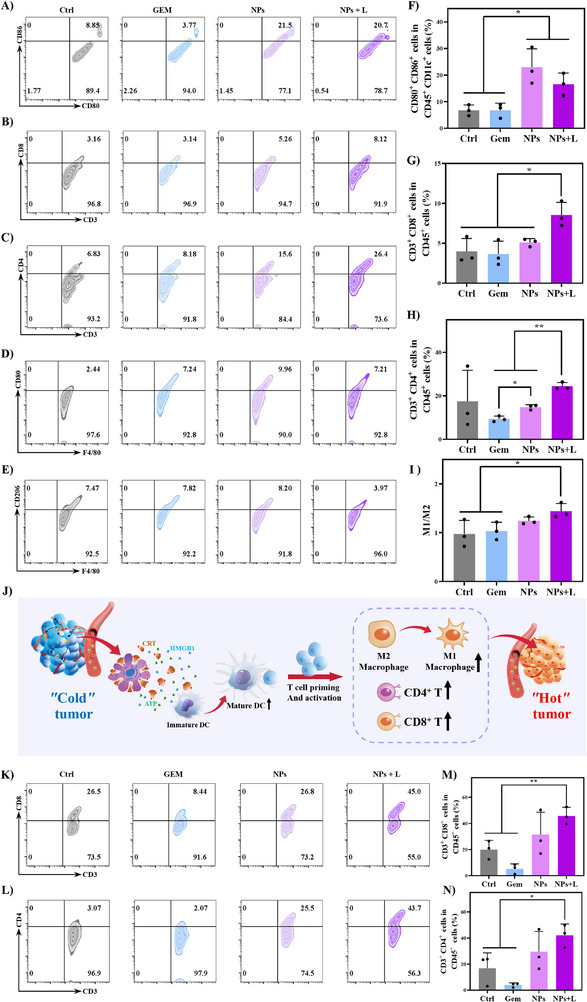
Regulation of the tumor immune microenvironment. (A) Representative flow cytometry plots and (F) quantitative analysis of mature dendritic cells (CD80^+^CD86^+^CD11c^+^) in tumors in the Balb/c tumor‐bearing mice after treatment with Saline as a control, GEM, NPs, or NPs + L. (B) Representative flow cytometry plots and (G) quantification of cytotoxic T cells (CD8^+^CD3^+^CD45^+^) in tumors. (C) Representative flow cytometry plots and (H) quantification of helper T cells (CD4^+^CD3^+^CD45^+^) in tumors. (D) Representative flow cytometry plots of M1 macrophages (CD80^+^F4/80^+^CD11b^+^), (E) representative flow cytometry plots of M2 macrophages (CD206^+^F4/80^+^CD11b^+^), and (I) the M1/M2 ratio in tumors. (J) Schematic diagram for modulation of tumor immune cell populations after NPs + L treatment. (K) Representative flow cytometry plots and (M) quantification of cytotoxic T cells (CD8^+^CD3^+^CD45^+^) in the spleen post‐treatment. (L) Representative flow cytometry plots and (N) quantification of helper T cells (CD4^+^CD3^+^CD45^+^) in the spleen post‐treatment.

Flow cytometric analysis was performed to analyze immune cell populations within both the tumor and spleen after different treatments (Figure 13). Within tumor microenvironment, the proportion of DCs (CD45^+^CD11c^+^CD80^+^CD86^+^) increases markedly from 6.76% in the control group to 16.57% after the NPs + L treatment, suggesting that antigen presentation is substantially enhanced. The activation of DCs may facilitate the priming of tumor‐specific T cells. Meanwhile, the fraction of CD8^+^ CTLs (CD45^+^CD3^+^CD8a^+^) rose from 3.98% to 8.55%, and the fraction of CD4^+^ helper T cells (CD45^+^CD3^+^CD4^+^) from 17.48% to 24.43%, supporting intensified effector T‐cell activation. Moreover, the M1/M2 macrophage ratio increases from 0.97 to 1.44, implying a phenotypic shift toward a pro‐inflammatory, tumoricidal state that helps strengthen antigen presentation and cytokine secretion.

A similar immune activation pattern is observed in the spleen. The percentage of CD8^+^ T cells increases from 20.00% to 45.77%, and CD4^+^ T cells from 16.79% to 42.10%, indicating that the NPs + L treatment not only remodels the local tumor immune landscape but also initiates a systemic immune response. Systemic expansion of effector T cells suggests that enhanced antigen presentation within tumors may promote peripheral T‐cell priming and proliferation. The concurrent activation of DCs, macrophages, and T cells suggest there may be a coordinated antitumor immune cascade: efficient antigen presentation by DCs triggers CD8^+^ and CD4^+^ T‐cell responses, leading to effective tumor clearance.

Collectively, these results support that the NPs + L therapy reprograms the immune milieu by augmenting antigen‐presenting activity, strengthening T‐cell responses, and driving macrophage polarization toward an inflammatory phenotype. Such comprehensive immune modulation can be attributed to ICD induced by the engineered nanoplatform, which integrates chemotherapeutic and photothermal effects to trigger the exposure and release of tumor‐associated antigens, promote DC maturation, and enhance subsequent T‐cell priming. These immune responses are the mechanistic basis for the potent and durable antitumor efficacy of the combined chemo‐photothermal therapy.

## 3 Conclusion

In summary, we constructed a peptide dendrimer‐based nanoplatform for mitochondria‐targeting cancer therapy that integrates chemotherapy, photothermal therapy, and immune modulation within a single system. The dendrimer scaffold allows controlled drug conjugation, promotes stable nanoparticle formation, and facilitates surface modification, collectively improving circulation stability and tumor accumulation of the resulting nanoparticles. After cellular uptake, the nanoparticles are preferentially localized to the mitochondria, where combined chemotherapeutic action and photothermal heating induce severe mitochondrial dysfunction, mitochondrial membrane depolarization, and apoptotic cell death. Importantly, these local effects are accompanied by ICD, including CRT exposure, HMGB1 release, and ATP release, which promotes dendritic cell maturation and enhanced T cell responses. In vivo studies have confirmed effective tumor accumulation, deep tissue penetration, and potent photothermal ablation, resulting in substantial inhibition of primary tumor growth and distinct reduction in lung metastasis as well as prolonged survival of tumor‑bearing mice. Flow cytometry analysis reveals an elevation in the infiltration of cytotoxic T lymphocytes, a shift in the macrophage phenotype toward a proinflammatory state. Encouragingly, there are no signs of systemic toxicity in histological or blood analyses after systemic administration of the nanoplatform. Collectively, these findings demonstrate that the synergistic interplay of chemotherapy, photothermal therapy, and immunogenic cell death not only directly eliminates tumor cells but also primes adaptive antitumor immunity, and it could be a promising approach to improving therapeutic efficacy against solid cancer.

## Conflicts of Interest

The authors declare no conflicts of interest.

## Supporting information




**Supporting File**: advs74589‐sup‐0001‐SuppMat.docx.

## Data Availability

The data that support the findings of this study are available from the corresponding author upon reasonable request.
