# Supplementary material for: A Mitochondria‐Specific Nanomedicine for Synergistic Chemo‐Photothermal Therapy and Immunogenic Activation Against Breast Cancer
Source: Adv Sci (Weinh). 2026 Feb 25;13(29):e22226. doi: 10.1002/advs.202522226 (PMC13205596; doi:10.1002/advs.202522226)
Supplement: Supplementary file 1 — Supporting File: advs74589‐sup‐0001‐SuppMat.docx. [file ADVS-13-e22226-s001.docx]

Supporting information

**A mitochondria-specific nanomedicine for synergistic chemo-photothermal therapy and immunogenic activation against breast cancer**

*Min Li, Jiangqi Feng, Junyang Zhuang, Qingguo Zhong, Yanda Li, Yanzhuo Lv, Shaoteng Huang, Xiangyu Huang, Mingbo Zhang, Xiaofeng Cai, Yuxin Wang, Wenping Chen, Zhenyu Duan, Zhou Chen, Kui Luo,^*^ Ning Li^*^*

M. Li, Prof. J. Zhuang, Y. Li, Y. Lv, S. Huang, X. Huang, M. Zhang, X. Cai, Y. Wang, W. Chen, Prof. Z. Chen, Prof. N. Li

Fujian Key Laboratory of Drug Target Discovery and Structural and Functional Research, School of Pharmacy, Fujian Medical University, Fuzhou 350122, China

^*^E-mail: ningli@fjmu.edu.cn (Prof. Li)

J. Feng

Department of Chemistry, University College London, London WC1H 0AJ, UK

Prof. Q. Zhong

School of Medicine, Fuzhou University, Fuzhou 350108, China

Prof. Z. Duan, Prof. K. Luo

Department of Radiology, Huaxi MR Research Center (HMRRC), Institution of Radiology and Medical Imaging, Frontiers Science Center for Disease-Related Molecular Network, State Key Laboratory of Biotherapy, West China Hospital, Sichuan University, Chengdu 610041, China. ^*^E-mail: luokui@scu.edu.cn (Prof. Luo)

**Keywords**: Amphiphilic peptide dendrimer, synergic therapies, transcytosis, mitochondria-specific, immunogenic cell death

**Content**

[1 Experimental Section 4](#_Toc212318971)

[1.1 Materials and measurements 4](#_Toc212318972)

[1.2 Synthesis and characterization of G3K-GFLG-Gem and IR825 5](#_Toc212318973)

[1.3 Preparation of G@IR825 NPs, G&HA NPs, and G@IR825&HA NPs 6](#_Toc212318974)

[1.4 Size, zeta potential, and morphology 6](#_Toc212318975)

[1.5 In-vitro release profile of G@IR825&HA NPs 7](#_Toc212318976)

[1.6 Cells and animals 7](#_Toc212318977)

[1.7 Cellular uptake 8](#_Toc212318978)

[1.8 Endocytic pathway of G@IR825&HA NPs 8](#_Toc212318979)

[1.9 In vitro cytotoxicity 8](#_Toc212318980)

[1.10 Studies on active targeting mediated by G@IR825&HA NPs 9](#_Toc212318981)

[1.11 Transcellular Transport of G@IR825&HA NPs 9](#_Toc212318982)

[1.12 3D Tumor spheroid penetration assays 10](#_Toc212318983)

[1.13 Mitochondrial colocalization study and mitochondrial membrane potential (MMP) analysis 11](#_Toc212318984)

[1.14 Inhibition of cell migration assay 11](#_Toc212318985)

[1.15 In-vitro evaluation of ICD induction ability 12](#_Toc212318986)

[1.16 Hemolytic assays 12](#_Toc212318987)

[1.17 Biodistribution studies and photothermal effect 13](#_Toc212318988)

[1.18 In vivo antitumor efficacy 13](#_Toc212318989)

[1.19 Analysis of survival and suppression of lung metastasis 14](#_Toc212318990)

[1.20 RNA sequencing (RNA-seq) analysis 14](#_Toc212318991)

[1.21 In vivo evaluation of tumor-infiltrating immune cells 15](#_Toc212318992)

[1.22 Statistical analysis 16](#_Toc212318993)

[2 Results 17](#_Toc212318994)

# **1 Experimental Section**

**1.1 Materials and measurements**

Boc-Lys (Boc)-OH, N-hydroxysuccinimide (NHS), and 1-Ethyl-3 (3-dimethylaminopropyl) carbodiimide hydrochloride (EDC·HCl) were purchased from Shanghai Gil Biochemical Co., Ltd (Shanghai, China). Gem·HCl was obtained from Adamas (Shanghai, China). Hyaluronic acid (HA, 40-100 kDa) was purchased from Shanghai Aladdin Biochemical Technology Co., Ltd (Shanghai, China). Chlorpromazine, amiloride hydrochloride, and Cy5-NHS (an organic small molecule dye) were purchased from GLPBIO (California, Montclair, USA). Papain and HAase were purchased from Sigma Aldrich (St. Louis, MO, USA). Roswell Park Memorial Institute (RPMI)-1640, and penicillin/streptomycin, Mitochondrial membrane potential assay kits with JC-1 were bought from Adamas (Shanghai, China). 2-(4-Amidinophenyl)-6-indolecarbamidinedihydrochloride (DAPI), Hoechst 33342, methylthiazolyldiphenyl-tetrazolium bromide (MTT), Calcein/PI cell viability assay kits, and ATP detection kits were bought from Beyotime Biotechnology (Shanghai, China). The mouse HMGB1 ELISA detection kit was purchased from Fine Biotech Co., Ltd (Wuhan, China). Rabbit anti-mouse CRT primary antibody and Cy5-labeled goat anti-rabbit IgG secondary antibody were obtained from Abcam Trading Co., Ltd (Cambridgeshire, UK).

Size and zeta potential were measured via a dynamic light scattering (DLS) particle size analyzer (Anton Paar, Graz, Austria). Morphological changes in nanoparticles were characterized by transmission electron microscopy (Tecnai G2, FEI Company, Oregon, USA). The temperature changes during photothermal therapy *in vitro* and *in vivo* were recorded by an infrared thermal imager (Teledyne FLIR, California, USA). Confocal imaging experiments were performed via confocal microscopy (Leica, SP5, Wetzlar, Germany). Flow semi-quantitative experiments were performed via a flow cytometry sorter (LSRFortessaX-20, New Jersey, USA). A UV-visible spectrophotometer (Shimadzu, UV-2600, Kyoto, Japan) and a fluorescence spectrophotometer (Agilent Technologies, California, USA) were used to detect the UV absorption spectrum and the fluorescence emission spectrum of the material, respectively.

For *in vitro* and *in vivo* experiments, “+/- L” indicated the presence or absence of laser irradiation in that group, and the light treatment groups were irradiated with a laser (Changchun Laser Optoelectronics Technology Co., Ltd Changchun, China) at a wavelength of 808 nm with a power of 1.0 W/cm^2^ for 5 min.

**1.2 Synthesis and characterization of G3K-GFLG-Gem and IR825**

The specific synthesis route was shown in Figure S1. G3K-alkyne was synthesized according to the previous work ^[1-3]^. G3K-alkyne (1.07 g, 100 µmol), N_3_-GFLG-GEM (979 mg, 1.25 mmol), and sodium ascorbate (267 mg, 1.35 mmol) were sequentially added into a flask. Under a nitrogen atmosphere, a mixture of H_2_O/DMSO at a volumetric ratio of 1:3 (100 mL) containing CuSO_4_·5H_2_O (170 mg, 0.68 mmol) was introduced into this flask. After stirring at 50^o^C for 2 days, the mixture was purified via a dialysis bag (MWCO = 3000). Dialysis was conducted against a stepwise diluted EDTA-2Na aqueous solution at a cold temperature (4^o^C). The solvent, catalyst, and unreacted raw material were removed through the dialysis method. After dialysis in ultrapure water, the product was collected and freeze-dried (1.3 g, a yield of 89.5%). As shown in **Figure S3**, the structure of G3K-GFLG-Gem was confirmed by the ^1^H NMR (600 MHz, DMSO), which exhibited proton signals at δ 8.32 ppm (t, 1H), 7.97–7.62 ppm (m, 5H), 6.97–6.62 ppm (m, 6H), 4.31–4.12 ppm (m, 3H), 3.93–3.75 ppm (m, 5H), 3.13–2.79 ppm (m, 12H), 1.70–1.03 ppm (m, 109H), and 0.96–0.77 ppm (m, 4H). High-resolution Q-TOF mass spectrometry further confirmed successful synthesis of G3K-GFLG-Gem (**Figure S4**). The Q-TOF MS (ESI⁺) spectrum revealed a prominent doubly charged sodium-adducted ion, with an m/z value of 1290.2024, which is in agreement with the calculated m/z of 1290.2103 for C_120_H_193_F_2_N_25_O_32_ ([M + Na]^2^⁺).

Click conjugation between G3K-Alkyne and N_3_-GFLG-Gem was verified by FT-IR spectroscopy (**Figure S5**). Compared with the characteristic alkyne (≡C–H) stretching vibration at 3300–3200 cm^-1^ for G3K-Alkyne and the azide (-N_3_) absorption band ~2120 cm^-1^ for N_3_-GFLG-Gem, both signals disappeared in the spectrum of G3K-GFLG-Gem, confirming successful CuAAC click reaction.

IR825 was synthesized according to a previously reported procedure ^[4-6]^ (Figure S2). Its ESI-MS analysis (Figure S6B) showed a major peak at an m/z of 823.4, which is in agreement with the calculated m/z value of 823.3 for C₅₄H₄₈BrClN₂O₄, [M-Br+H]⁺. The purity of IR825 was determined to be 92% by HPLC (**Figure S6C**). Fluorescence emission spectroscopy revealed an emission maximum peak at approximately 615 nm (**Figure S6D**).

**1.3 Preparation of G@IR825 NPs, G&HA NPs, and G@IR825&HA NPs**

The preparation process was shown in Scheme 1 (A). Nanoparticles were prepared via the nanoprecipitation method. In brief, NH_2_-G3K-GFLG-Gem (4.7 mg) and IR825 (1.4 mg) were completely dissolved in DMSO (1 mL) under sonication for 30 min. The mixture was added dropwise at a rate of one drop per minute (5 µL per drop) to ultrapure water (10 mL) under rapid stirring. After 10 h, a clear and homogeneous green solution was obtained. Then, the HA (40–100 kDa) solution that was pre-swollen completely was poured to the mixture, and the mixture was stirred for 15 min. Finally, after removal of DMSO and unencapsulated free materials through dialysis, the G@IR825&HA nanoparticles were obtained after freeze-drying. Meanwhile, G@IR825 NPs and G&HA NPs were prepared by similar protocols.

**1.4 Size, zeta potential, and morphology of nanoparticles**

The nanoparticles were assayed through DLS and TEM. The nanoparticles were dissolved in ultrapure water and sonicated for 30 min to ensure complete dispersion before measurements. The size and zeta potential of samples (100 µg/mL) were detected via a DLS particle size analyzer. Each measurement was performed in triplicate. The freshly prepared NPs aqueous solution (100 µg/mL) was applied onto a 150-mesh carbon-coated copper grid. The excess solution was carefully removed from the edge of the copper grid with a filter paper and dried naturally at room temperature, and no additional staining was applied. Finally, the NPs were observed under the TEM. In addition, the hydrodynamic size and zeta potential of the NPs were continuously monitored over a period of seven days to evaluate their colloidal stability. Meanwhile, the NPs aqueous solution after one week of storage was subjected to TEM analysis using the same preparation procedure to examine morphological changes of the NPs.

**1.5 *In-vitro* release profile of G@IR825&HA NPs**

Gemcitabine (Gem) release from the G@IR825&HA nanoparticle was assessed in a buffer solution with or without papain. Papain has a similar activity as lysosomal cathepsin B. The same volume of a papain and HAase mixture solution and a glutathione solution were prepared in the McIlvaine's buffer (10 mM). Both solutions were mixed homogeneously, and the mixture was placed in a 37^o^C water-bath. The G@IR825&HA NPs were added to the preheated mixture (containing papain and HAase, pH = 5.4) and incubated in a thermostatic water-bath shaker for 36 h. Meanwhile, the group incubated in a PBS buffer (pH = 7.4, 6.5 or 5.4) without papain and HAase was set as the control group. At predetermined time points, a fixed volume of the buffer was withdrawn from the centrifuge tube and dissolved in a methanol solution (HPLC grade). After vortexing and filtering, the Gem content released from the NPs was determined by RP-HPLC with a UV detector at 275 nm with column specifications (SHIMADZU, C8, 4.6 × 150 mm, 5 μm). The gradient program from 2 to 98% of Buffer B within 20 min was applied at a flow rate 1.0 mL/min (Buffer A: deionized water with 0.1% TFA, Buffer B: acetonitrile containing 0.1% TFA).

**1.6** **Cells and animals**

The 4T1 cell line (murine breast cancer cell) was purchased from the National Collection of Authenticated Cell Cultures (Shanghai, China; Catalog No. SCSP-5056, Order No. 261010, placed on August 14, 2023, and received on August 23, 2023). The cell line was cultured in RPMI 1640, supplemented with 10% (v/v) fetal bovine serum (Umedium, He Fei, China) and 1% (v/v) penicillin/streptomycin. Human umbilical vein endothelial cells (HUVECs) were purchased from iCell Bioscience Inc (Shanghai, China; Catalog No. iCell-h110) and cultured in DMEM. The cells were cultured in a humidified incubator (5% CO_2_/95% air atmosphere at 37^o^C), and was confirmed to be free of mycoplasma contamination. Female Balb/c mice (20 ± 2 g, 6-8 weeks old) were used to establish tumor models. The animals were purchased from Wu's Laboratory Animal Co., Ltd (Fujian, China). They were randomly divided, scientifically fed by implementing the principles of animal welfare, and acclimatized to the rearing environment for about one week before experiments. All animal experiments were in accordance with national regulations and approved by the Ethics Committee of animal experiments (approval number: IACUC FJMU 2022-0035).

**1.7 Cellular uptake**

4T1 cells were seeded in glass-bottom dishes, washed, and then incubated with the medium containing 10 μg/mL G@IR825&HA NPs for different durations (0, 1, 2, 4, and 6 h). After incubation, the cells were washed, stained with Hoechst 33342 and immediately observed by CLSM. Similarly, to investigate the effect of PTT treatment on cellular uptake of NPs, the 4T1 cells were incubated with Cy5-labeled NPs for 4 h, the cells were irradiated with or without laser irradiation and then incubated for another 4 h. Cells were observed under the CLSM and quantification of the fluorescence signal in cells was performed via the FCM.

**1.8** **Endocytic pathway of G@IR825&HA NPs**

To reveal the endocytic pathway involved in NPs internalization, 4T1 cells were seeded into [12](javascript:;)-well plates at a density of 2 × 10^5^ cells per well and incubated for 24 h. These cells were preincubated with five specific endocytic inhibitors (chlorpromazine (Chl, 60 µM), wortmannin (Wor, 5 µM), or nystatin (Nys, 20 µM)) in a serum-free medium or at 4 ^o^C for 2 h. The medium was then replaced by a fresh serum-free medium containing the NPs at an IR825 concentration of 25 μg/mL and further incubated for 6 h at 37^o^C. Meanwhile, the cells in the 4^o^C group were incubated for 6 h at 4^o^C. Finally, the cells were washed thrice with PBS, digested and collected, the fluorescence intensity of IR825 (Ex = 580 nm, Em = 615 nm) was measured via the FCM.

**1.9 *In vitro* cytotoxicity**

4T1 cells were seeded in a 96-well plate at a density of 5 × 10^3^ cells per well. After 24 h, the medium was replaced by G@IR825&HA NPs at a concentration gradient. After 4 h incubation of NPs, the cells were irradiated with a laser. Cells were transferred to the incubator for another 20 h before MTT assays. Meanwhile, the cells treated with NPs (- L) and free Gem were used as control groups.

To observe live/dead cells induced by G@IR825&HA NPs, 4T1 cells were seeded in glass-bottom cell culture dishes. After incubation with different treatments (PBS +/- L, free Gem, IR825 +/- L, and NPs +/- L) for 4 h, a fresh medium was replaced, and cells were incubated overnight. The cells were stained with propidium iodide (PI) and calcein AM for observation under the CLSM.

**1.10** **Active targeting mediated by G@IR825&HA NPs**

The active targeting capability of G@IR825&HA NPs was validated by competitive inhibition experiments. Specifically, the 4T1 cells were seeded in a 96-well plate at a density of 5 × 10^3^ cells per well. After 24 h, the cells were pretreated with a completely pre-swollen HA sterile solution at different concentrations (0, 5, and 7 mg/mL) for 2 h. These cells were washed and incubated with the NPs solution for 24 h. The MTT assay was used to evaluate the IC50 (half maximal inhibitory concentration) value of NPs. In addition, 4T1 cells were seeded into glass-bottom dishes or 24-well plates, and cellular uptake of NPs by pretreated cells with the HA solution at different concentrations was detected by confocal microscopy and flow cytometry.

**1.11 Transcellular transport of G@IR825&HA NPs**

To examine transcellular transport of G@IR825&HA NPs, a sequential “infection” assay was performed. Briefly, 4T1 cells were seeded in glass-bottom dishes at a density of 1 × 10^5^ cells per dish in 1 mL of a fresh complete medium and cultured for 24 h. These cells were designated as the first batch. They were incubated with G@IR825&HA NPs or free IR825 at an equivalent IR825 concentration of 35 μg/mL for 4 h, followed by laser irradiation (808 nm, 1.0 W/cm^2^, 5 min). After treatment, the cells were washed with PBS and imaged under the confocal microscopy. Subsequently, the cells were cultured in 1 mL of a fresh medium for 12 h. 0.8 mL of the conditioned medium was collected and mixed with a fresh medium to treat a second batch of naive 4T1 cells for an additional 12 h. After incubation, the second batch of cells was washed with PBS, stained with Hoechst 33342, and immediately imaged under the CLSM. This process was repeated to evaluate nanoparticle transfer across multiple cellular layers.

To further simulate intercellular transport of G@IR825&HA NPs, a transwell-based co-culture system was employed. 4T1 cells were seeded onto the upper chamber of a transwell (polyester membrane, pore size: 0.4 μm, Corning) at a density of 3 × 10^4^ cells per chamber and incubated for 24 h. These cells were treated with G@IR825&HA NPs or free IR825 (35 μg/mL) for 4 h, followed by laser irradiation (808 nm, 1.0 W/cm^2^, 5 min). Meanwhile, 4T1 cells (5 × 10^4^) were pre-seeded on sterile glass coverslips in 12-well plates. The coverslips were transferred into the lower chamber. Treated cells in the upper chamber were co-cultured with cells in the low chamber for 12 h. After incubation, the cells on coverslips in the lower chamber were washed with PBS, fixed with 4% paraformaldehyde, stained with DAPI, and imaged under the CLSM.

**1.12** **3D Tumor spheroid penetration assays**

*In vitro* drug penetration was observed using a 3D tumor spheroids model, and multicellular tumor spheroids (MTSs) were prepared in 2% agarose gel solution. In brief, a 4T1 cell suspension at a density of 1 × 10^4^ cells per well was seeded into 96-well plate containing 2% coagulated agarose gel and cultured for about 3 days. When the size of the 4T1 MTSs became around 250 µm, they were transferred to glass-bottom dishes and incubated with NPs at an IR825 concentration of 40 μg/mL for predetermined time intervals. They were washed with PBS and fixed with 4% paraformaldehyde (PFA). IR825 signals were scanned every 10 µm from the top to the middle layer under the CLSM.

In addition, we explored the impact of photothermal therapy (PTT) and hyaluronidase (HAase) treatment on the permeation efficacy of nanoparticles (NPs) within multilayered tumor spheroids (MTSs). The MTSs were incubated with NPs for 12 h and irradiated with or without a laser. Meanwhile, the MTSs were pretreated with or without HAase at a concentration of 150 IU/mL for 4 h prior to the evaluation of NP penetration into MTSs.

**1.13** **Mitochondrial colocalization and mitochondrial membrane potential (MMP) analysis**

To assess sub-cellular localization of G@IR825&HA NPs within mitochondria, 4T1 cells were seeded in glass-bottom dishes at a density of 1 × 10^5^ cells per well and cultured for 24 h. After rinsing with PBS, cells were incubated with G@IR825&HA NPs at an equivalent IR825 concentration of 35 µg/mL for 4 h. These cells were washed and stained with Mito-Tracker (200 µL, 200 μM) at 37^o^C for 45 min. They were washed three times with cold PBS, stained with Hoechst 33342, and immediately observed under the CLSM. The confocal image analysis platform and the Image J software were used to analyze the level of co-localization.

To investigate mitochondrial damage by NPs, mitochondrial membrane potential analysis was performed. Briefly, the 4T1 cells were seeded in glass-bottom dishes at a density of 1 × 10^5^ cells per well and incubated for 24 h. The incubation media were replaced with fresh media for different treatments (PBS +/- L, IR825 +/- L, free Gem, and NPs +/- L (100 μg/mL)) for 4 h. After continuous incubation for another 20 h, the cells were incubated with JC-1 (2 μM) for 20 min, washed, replaced with PBS, and finally observed by the CLSM at an excitation wavelength of 488 nm for JC-1. The detection of the experiment was performed according to the manufacturer’s instructions.

**1.14** **Inhibition of cell migration assay**

Cell migration was evaluated with cell scratch and transwell cell migration assays (8 μm, Corning Life Sciences). For the cell scratch experiment, the 4T1 cells were seeded into 6-well plates at a density of 1 × 10^6^ cells per well and incubated for 24 h. A sterilized pipette tip was used to make a straight scratch in the wells, and the cells were washed three times with sterile PBS to remove the exfoliated cells to ensure a distinctively visible gap after streaking. After the cells were incubated with RPMI-1640 (containing 1% FBS) containing G@IR825&HA NPs at different concentrations, cell migration was observed under an inverted fluorescence microscope.

For the transwell cell migration assay, the 4T1 cells were pretreated with different treatments (PBS +/- L, IR825 +/- L, and NPs +/- L), and seeded into the transwell upper chamber. Meanwhile, RPMI-1640 (containing 1% FBS) at an appropriate volume was added to the lower chamber. After 24 h, the migrated cells were fixed and stained with 4% PFA and 1% crystal violet. They were observed under an inverted fluorescence microscope. Crystal violet was dissolved in a 33% acetic acid solution and the absorbance value was measured at 570 nm.

**1.15** ***In-vitro* evaluation of ICD induction**

***Exposure of Calreticulin (CRT):*** 4T1 cells were seeded on a glass-bottom dish at a density of 1 × 10^4^ cells per well overnight and treated with different formulations (PBS +/- L, free Gem, IR825 +/- L, and NPs +/- L) for 4 h. After 20 h, the cells were washed three times with cold PBS and fixed in 4% PFA for 20 min. These cells were washed with cold PBS and incubated with rabbit anti-mouse CRT primary antibody at 4^o^C for 1 h (1: 500). They were washed and incubated with the Cy5-conjugated secondary antibody (1: 500) for 30 min at room temperature. They were stained with DAPI and examined by CLSM.

***Release of High Mobility Group Box 1 Protein (HMGB1):*** Extracellular HMGB1 secretion was detected by a HMGB1 ELISA kit. Briefly, 4T1 cells were seeded on a 12-well plate at a density of 2 × 10^5^ cells per well overnight and treated with different formulations (PBS +/- L, free Gem, IR825 +/- L, and NPs +/- L) for 4 h. After 20 h, the detection of HMGB1 was performed according to the standard protocol provided by the manufacturer.

***Secretion of Adenosine Triphosphate (ATP):*** Extracellular secretion of ATP was assessed via an ATP assay kit. The cellular treatment was the same as that for the detection of HMGB1. The cell culture supernatant was collected, and the concentration of ATP was determined via the ATP assay kit according to manufacturer’s instructions.

**1.16** **Hemolytic assays**

The hemolytic experiment was carried out to evaluate hemocompatibility of nanoparticles. The G@IR825&HA NPs solution at different concentrations was added to a 5% murine red blood cell (RBC) suspension. Triton-X-100 was used as a positive control and saline was used as a negative control. All samples were incubated at 37^o^C for 2 h on a shaker. After these samples were centrifuged for 5 min (4^o^C, 3500 rpm), supernatants were collected, and their absorbances were measured at 545 nm. The hemolysis rate was evaluated from the formula:

Hemolysis (%) = (Absorbance in the experimental group - Absorbance in the negative control) / (Absorbance of the positive control - Absorbance of the negative control) × 100%

**1.17** **Biodistribution studies and photothermal effects**

In the biodistribution study, the 4T1 tumor-bearing Balb/c female mice were injected with G@IR825&HA NPs at an IR825concentration of 8 mg/kg through the tail vein. The fluorescence imaging was performed via a near-infrared fluorescence *in vivo* imaging system at the time points prespecified (0, 8, 12, 24, and 36 h). The main organs including the heart, liver, spleen, lung, kidney, and tumor were harvested for *in-vitro* imaging.

In the *in vivo* photothermal effect study, tumor-bearing mice was injected with G@IR825&HA NPs at an IR825 concentration of 8 mg/kg. After 12 h, the mice were irradiated with a laser. Temperature changes and infrared thermal images were recorded real-time via an infrared thermal imager.

**1.18 *In vivo* antitumor efficacy**

To evaluate the *in vivo* therapeutic effect of G@IR825&HA NPs, a subcutaneous transplantation tumor model was established. When the tumor volume reached around 100 mm^3^, the 4T1 tumor-bearing Balb/c female mice were divided into six groups randomly (n = 3 per group). They were intravenously injected with saline, free Gem, or G@IR825&HA NPs at an equivalent Gem concentration of 5 mg/kg on day 1. A half of the mice receiving each injection were subjected to laser treatment, and the group were denoted as the + L group. Laser irradiation was performed at 12 h post-injection. The tumor volume and the mice body weight were monitored every other day for a period of 19 days. The tumor volume was calculated from the formula: Tumor volume = 1/2 × length × width^2^, and the relative tumor volume was defined as: Relative tumor volume (%) = V/V_0_×100%. To quantitatively evaluate the tumor growth inhibition efficacy of different treatments, TGI was calculated as: TGI = (1-(the mean tumor weight of the treatment group)/(the mean tumor weight of the control group)) × 100%.

To evaluate the hemocompatibility of G@IR825&HA NPs, blood was collected for hematological analysis on day 19. Meanwhile, the major organs were excised, followed by hematoxylin and eosin (H&E) staining. Moreover, tumors were stained with TUNEL, CD31, and Ki67 to evaluate the therapeutic effect of G@IR825&HA NPs.

**1.19 Analysis of survival and suppression of lung metastasis**

To assess the impact of G@IR825&HA NP treatment on the overall survival of the mice, the survival experiment was performed. Briefly, the tumor volume and the body weight were consistently measured every other day. When the tumor size reached 2000 mm^3^, the mice were considered dead according to animal welfare guidelines. The survival time of the mice was recorded, and the lung tissues were removed and fixed with 4% PFA for 24 h. The number of metastatic nodules in lung tissues were counted and photographed. Finally, the lung tissues were embedded in paraffin and analyzed by HE staining.

**1.20** **RNA sequencing (RNA-seq) analysis**

4T1 cells were seeded into 24-well plates at a density of 1.5 × 10^5^ cells per well and cultured overnight. The cells were then incubated with the free drug, NPs, or PBS (control) for 4 h. Subsequently, the NPs + L group was irradiated with an 808 nm laser (1 W/cm^2^) for 5 min. After an additional 8 h of incubation, cells were harvested. The cells were washed three times with PBS, trypsinized, and collected by centrifugation (4 ^o^C, 200 x g, 5 min). Cell pellets were lysed using 1 mL of the TRIzol reagent for every 5 × 10^6^ cells. The lysates were snap-frozen in liquid nitrogen for 30 min. RNA extraction, reverse transcription, library construction, and sequencing were conducted by Shanghai Majorbio Bio-Pharm Biotechnology Co., Ltd. (Shanghai, China) following the manufacturer’s protocol (Illumina, San Diego, CA, USA). Volcano plots, heatmaps, GO enrichment analysis, KEGG enrichment analysis, and Reactome pathway analysis were performed on the Majorbio Cloud Platform (<https://cloud.majorbio.com/page/tools.html>).

**1.21** ***In vivo* evaluation of tumor-infiltrating immune cells**

To evaluate the effect of G@IR825&HA NPs on the number of tumor-infiltrating immune cells *in vivo*, a subcutaneous 4T1 tumor model was established. When the tumor volume reached approximately 200 mm^3^, the mice were randomly divided into four groups (n = 3 per group). The mice received intravenous injections of saline, free Gem or G@IR825&HA NPs (5 mg/kg of Gem). A half of the NP-treated mice were subjected for irradiation, and the tumors were exposed to an 808 nm laser for 5 min at 12 h post-injection.

Seven days after treatment, tumors and spleens were excised, mechanically dissociated, and passed through a 70 μm nylon strainer to generate single-cell suspensions. The spleen samples were pretreated with a cold red blood cell lysis buffer for 10 min. Cells from both tumors and spleens were centrifuged at 3,500 rpm for 5 min at 25^o^C and fixed with 4% paraformaldehyde (PFA) for 15 min. After centrifugation, cells were resuspended in 1% BSA and 1% PFA to minimize nonspecific binding. Single-cell suspensions were subsequently stained with antibodies and analyzed by flow cytometry.

**1.22 *In vivo* biosafety assay on normal healthy mice**

To evaluate systemic safety of different formulations, healthy mice were randomly divided into three groups (n = 3 per group): Control (saline), free Gem, and NPs + L. All treated groups were *i.v.* injected an equivalent Gem dose of 5 mg/kg. For the NPs + L group, NIR laser irradiation (1 W/cm^2^, 5 min) was applied at 12 h post-injection at a similar site as that for tumor implantation in the *in vivo* antitumor efficacy experiments. After nine days post-treatment, mice were euthanized, and blood samples were collected for hematological analysis. Major organs, including the heart, liver, spleen, lung, and kidney, were harvested, fixed, and subjected to histological examination by hematoxylin and eosin (H&E) staining to assess treatment-induced toxicity.

**1.23 *In vivo* antitumor assay in a bilateral tumor model**

To evaluate systemic antitumor immune responses, a bilateral tumor model was established. Briefly, 4T1 cells (1 × 10^6^) were subcutaneously inoculated into the right flank of female BALB/c mice to establish the primary tumor. When the primary tumors reached an appropriate size of around 50–100 mm^3^, the mice were randomly assigned to four different treatment groups treated with saline as a control, free drug Gem, NPs, NPs + L at an equivalent Gem dose of 5 mg/kg. For the NPs + L group, NIR laser irradiation (1 W/cm^2^, 5 min) was applied locally to the primary tumor site at 12 h post-injection.

After 6 days post-treatment, 4T1 cells (5 × 10^5^) were inoculated into the contralateral flank to establish distant tumors without any treatment. Tumor growth of both primary and distant tumors was monitored throughout the experimental period by measuring the tumor length and width, and the tumor volume was calculated via the same formula in Section 1.18. At the end of the experiment, mice were sacrificed, and both primary and distant tumors were excised and weighed for analysis.

**1.24 Statistical analysis**

Quantitative data were presented as mean ± SD (n ≥ 3). Statistical analysis of the data was performed with one-way analysis of variance (ANOVA). Data are expressed as the mean ± SD (^*^*p* < 0.05, ^**^*p* < 0.01, ^***^*p* < 0.001, and ^****^*p* < 0.0001). Statistical significance was denoted as “not significant (ns)” when *p* > 0.05.

# **2 Results and discussion**

## **2.1 Stability evaluation of G@IR825&HA nanoparticles**

The stability of G@IR825&HA nanoparticles in an aqueous buffer was evaluated by monitoring their hydrodynamic size and zeta potential over a one-week period. As shown in **Figure S20**, the DLS results indicate that the nanoparticles exhibited moderate variations, and there was no statistical difference in the size throughout the observation period. Meanwhile, the zeta potential maintained a relatively stable level with minor fluctuations during storage, suggesting that the surface charge properties were preserved. In addition, TEM images of the nanoparticles prepared from aqueous dispersion after 7 days showed no apparent morphological changes compared with freshly prepared samples. These results indicate that G@IR825&HA nanoparticles displayed colloid stability over 7 days.

## **2.2 *In vivo* biosafety evaluation on normal healthy mice**

To evaluate *in vivo* biosafety of different formulations, including saline (control), free Gem, and G@IR825&HA NPs, normal healthy mice were subjected to histological and hematological analyses. As shown in **Figure S21**A, hematoxylin and eosin (H&E) staining images of major organs (including the heart, liver, spleen, lung, and kidney) revealed no apparent pathological abnormalities or tissue damage in all treatment groups, indicating there was no distinct observable organ toxicity after administration of the above formulations. In addition, routine blood biochemical and hematological parameters were analyzed to assess systemic safety of these formulations (**Figure S21**B). Slight fluctuations were observed in certain indices in the treated groups, including a modest decrease in hematocrit (HCT) and the mean platelet volume (MPV), however, the majority of hematological parameters remained within a normal physiological range and they were comparable to those of the control group. These variations in these parameters did not result in severe hematological toxicity. Collectively, these results suggest that G@IR825&HA NPs exhibited a favorable *in vivo* biosafety profile at the tested dose, supporting the applicability of the nanoparticle formulation for subsequent therapeutic studies.

## **2.3 Validation of systemic antitumor immunity in the bilateral tumor model**

In this study, we employed a bilateral tumor model to evaluate systemic antitumor immune responses elicited by the therapeutic regimen. As shown in **Figure S22**, the NPs + L group exhibited pronounced inhibition of primary tumor growth, and complete tumor regression was observed in a subset of the treated mice (**Figure S22**A and E). Notably, upon subsequent inoculation of tumor cells at a contralateral site, the mice receiving the NPs + L treatment displayed the lowest tumor incidence rate and the slowest tumor growth rate compared to other treatment groups (**Figure S22**B and F). Consistently, both primary and distant tumor weights were significantly reduced in the NPs + L group (**Figure S22**C and D), which was corroborated by the tumor photographs (**Figure S23**). These bilateral tumor treatment outcomes were consistent with the enhanced antitumor efficacy observed in the unilateral tumor model (Figure 10), the prolonged survival benefits (Figure 11), and the immune profiling results obtained by flow cytometry (Figure 13). Taken together, our results indicate that the combination of nanoparticle-mediated chemotherapy and photothermal therapy not only achieved effective local tumor ablation but also elicited a systemic antitumor effect to realize effective suppression of distant tumor establishment and growth in a bilateral tumor model.

**Figure S1.** The synthesis route of the G3K-GFLG-Gem prodrug.

**Figure S2.** The synthesis route of the photothermal agent IR825.

**Figure S3.** ^1^H-NMR spectrum of G3K-GFLG-Gem in DMSO-*d*_6_ (600 MHz).

**Figure S4.** Q-TOF-MS of G3K-GFLG-Gem (in a positive ion mode).





**Figure S5.** Infrared spectra of raw materials (G3K-Alkyne and N_3_-GFLG-Gem) and products (G3K-GFLG-Gem) before and after click reaction.

**Figure S6.** A) ^1^H NMR spectrum of IR825 in DMSO-*d*_6_ (600 MHz). B) ESI-MS of IR825 (in a positive ion mode).C) HPLC chromatogram of IR825. D) Fluorescence spectrum of IR825.


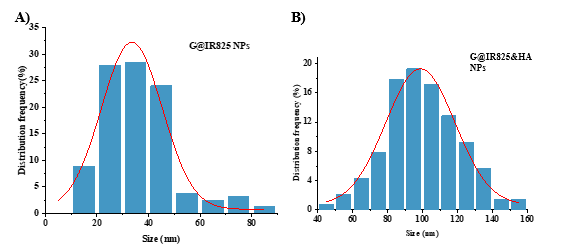


**Figure S7.** The size distribution of A) G@IR825 and B) G@IR825&HA nanoparticles derived from TEM images.

**Figure S8.** The FT-IR spectrum of HA and G@IR825@HA NPs.

**Figure S9.** A) Heating curves of the IR825 solution at the same concentration under 808 nm laser irradiation at different powers (0.5, 1.0, and 1.5 W/cm^2^). B) Heating curves of pure water and IR825 solutions with different concentrations (40, 100, and 200 μg/mL) under 808 nm laser irradiation (0.5 W/cm^2^, 5min). C) Photothermal stability of IR825 (40 μg/mL). The photo-to-thermal conversion efficiency of D) free IR825 and E) G@IR825&HA at an equivalent concentration of 20 μg/mL.

**Figure S10.** CLSM images of 4T1 cells treated with G@IR825 without HA coating for 0, 1, 2, 4, and 6 h. Blue fluorescence for the nucleus stained by DAPI and red fluorescence for IR825 in G@IR825&HA NPs. Scale bar: 25 µm.

**Figure S11**. Statistical quantification of the flow cytometry data in Figure 4B to evaluate the active targeting capability of G@IR825&HA NPs. Data are expressed as the mean ± SD (**p* < 0.05, ***p* < 0.01, ****p* < 0.001).

**Table S1. The IC50 values of different treatments**

| **Groups** | **IC50** |
| --- | --- |
| G@IR825&HA + 0 mg/mL HA | 22.54 μg/mL |
| G@IR825&HA + 5 mg/mL HA | 49.19 μg/mL |
| G@IR825&HA + 7 mg/mL HA | 70.64 μg/mL |

**Figure S12.** Alterations in A) the size and B) the zeta potential of NPs following *in vitro* enzymatic incubation.

**Figure S13.** *In vitro* cell viability of A) 4T1 cells treated with IR825 at various concentrations and B) HUVEC cells treated with Gem, NPs, and NPs with laser at various concentrations via the MTT assay.


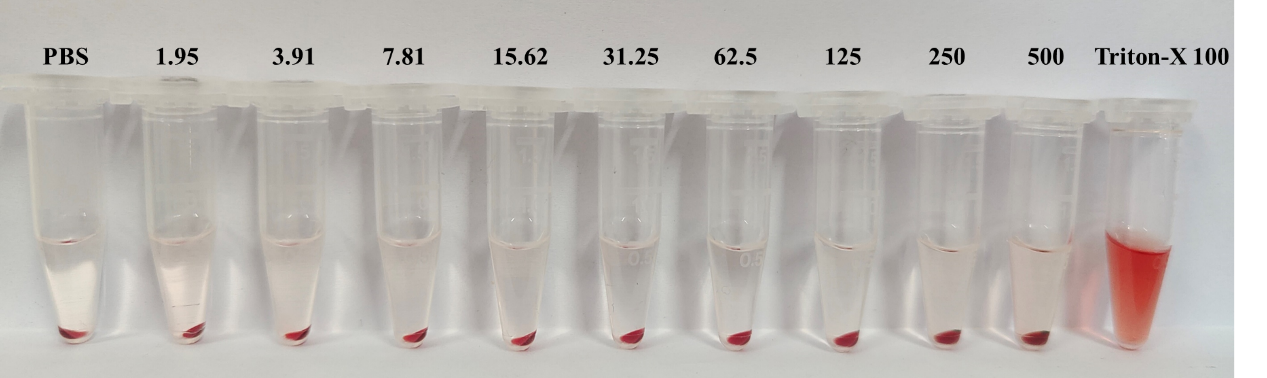


**Figure S14.** *In vitro* hemolysis tests. NPs were incubated with different concentrations of NPs. The PBS group and the Triton-X100 group were used as negative and positive control groups, respectively.


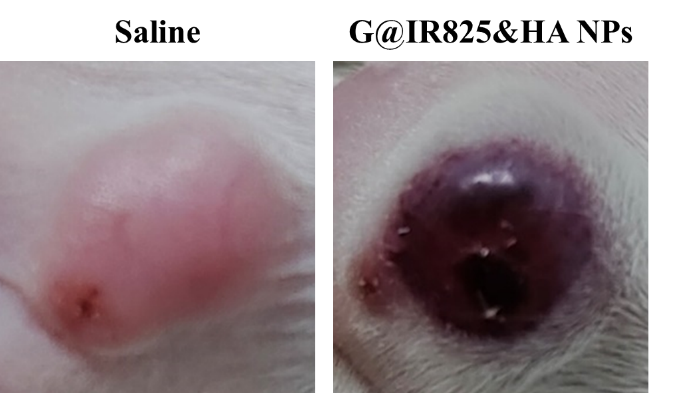


**Figure S15.** Photo images for tumors in the Saline and NPs + L treatment groups.

**Figure S16.** Blood routine analysis for the mice after different treatments at the end of the treatment period (n = 3).


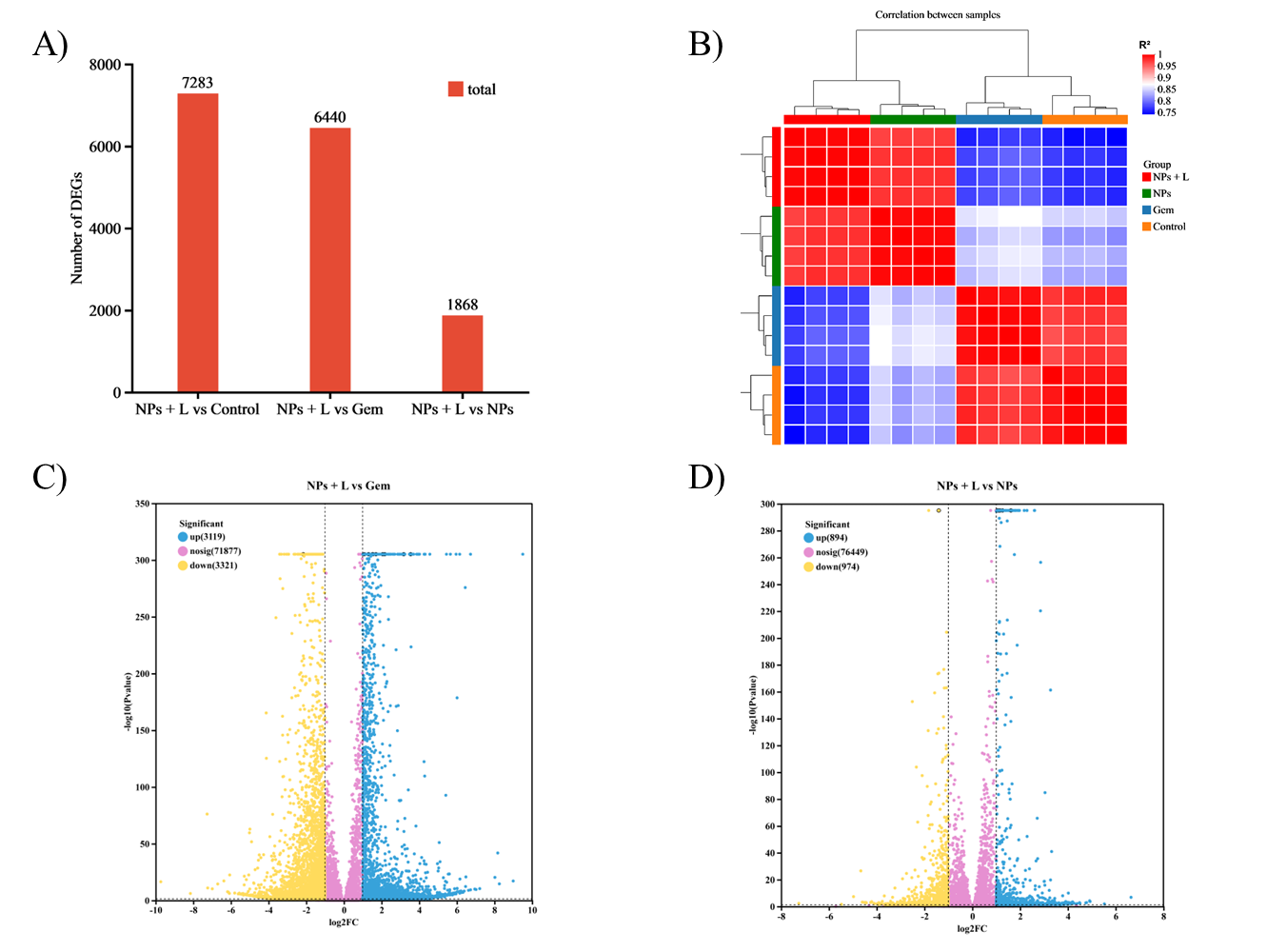


**Figure S17.** RNA-seq analysis. A) Comparison of the total number of differentially expressed genes between different groups and the NPs + L group. B) Correlation Analysis. C-D) Volcano plots for DEGs in the NPs + L group versus the Gem and NPs group. “Nosig” for genes without significant differential expression.

**Figure S18.** UV-vis spectra of N_3_-GFLG-Gem, G3K-A, and G3K-GFLG-Gem.

**Figure S19.** Photographs of NH_2_-G3K-GFLG-Gem (middle), NH_2_-G3K-GFLG-Gem with laser (left), and G@IR825&HA NPs (right) in water.

**Figure S20.** A) The hydrodynamic diameter and B) zeta potential of the NPs monitored by DLS over seven days. C) TEM image of the NPs solution after one week of storage, scale bar: 200 nm.

**Figure S21.** A) Histological and B) hematological analysis for different organs of health mice treated with control (saline), free drug Gemcitabine (Gem) and NPs. Scale bar: 100 μm. Data are expressed as the mean ± SD (n = 3, **p* < 0.05).

**Figure S22.** Antitumor efficacy in a bilateral tumor model. A) Growth curves of primary tumors during the treatment period and B) growth curves of distant tumors after contralateral tumor inoculation. Weights of C) primary tumors and D) distant tumors at the experimental endpoint. Tumor growth curves of individual mice in each group for primary (E) and distant (F) tumors. Data are expressed as the mean ± SD (n = 3, **p* < 0.05, ***p* < 0.01, and ****p* < 0.001).

**Figure S23.** Images of excised A) primary and B) distant tumors from different treatment groups at the end of the experiment.

**References**

[1] X. Huang, Z. Shen, Q. Deng, C. Zhao, X. Cai, S. Huang, M. Zhang, Y. Lv, D. Yang, J. Zhuang, N. Li, Chem. Eng. J. **2025**, *505*, 159380.

[2] S. Huang, X. Cai, M. Zhang, W. Yao, Q. Fang, Y. Dong, Y. Zhang, Y. Chen, J. Zhuang, N. Li, Biomacromolecules. **2025**, *26*, 634054.

[3] X. Song, H. Cai, Z. Shi, Z. Li, X. Zheng, K. Yang, Q. Gong, Z. Gu, J. Hu, K. Luo, Adv. Sci. **2024**, *11*, 2306230.

[4] L. Cheng, W. He, H. Gong, C. Wang, Q. Chen, Z. Cheng, Z. Liu, *Adv. Funct. Mater.* **2013**, *23*, 5893-5902.

[5] H. Gong, Z. Dong, Y. Liu, S. Yin, L. Cheng, W. Xi, J. Xiang, K. Liu, Y. Li, Z. Liu, Adv. Funct. Mater. **2014**, *24*, 6492-6502.

[6] L. Shao, Q. Li, C. Zhao, J. Lu, X. Li, L. Chen, X. Deng, G. Ge, Y. Wu, Biomaterials. **2019**, *194*, 105-116.
